# Have Synergies Between Nitrogen Deposition and Atmospheric CO_2_ Driven the Recent Enhancement of the Terrestrial Carbon Sink?

**DOI:** 10.1029/2018GB005922

**Published:** 2019-02-10

**Authors:** Michael O'Sullivan, Dominick V. Spracklen, Sarah A. Batterman, Steve R. Arnold, Manuel Gloor, Wolfgang Buermann

**Affiliations:** ^1^ Institute for Climate and Atmospheric Science, School of Earth and Environment University of Leeds Leeds UK; ^2^ School of Geography University of Leeds Leeds UK; ^3^ Institute of Geography Augsburg University Augsburg Germany; ^4^ Institute of the Environment and Sustainability University of California, Los Angeles Los Angeles CA USA

**Keywords:** carbon‐nitrogen interactions, terrestrial carbon sink, nitrogen deposition, CO_2_ fertilization, land surface models

## Abstract

The terrestrial carbon sink has increased since the turn of this century at a time of increased fossil fuel burning, yet the mechanisms enhancing this sink are not fully understood. Here we assess the hypothesis that regional increases in nitrogen deposition since the early 2000s has alleviated nitrogen limitation and worked in tandem with enhanced CO_2_ fertilization to increase ecosystem productivity and carbon sequestration, providing a causal link between the parallel increases in emissions and the global land carbon sink. We use the Community Land Model (CLM4.5‐BGC) to estimate the influence of changes in atmospheric CO_2_, nitrogen deposition, climate, and their interactions to changes in net primary production and net biome production. We focus on two periods, 1901–2016 and 1990–2016, to estimate changes in land carbon fluxes relative to historical and contemporary baselines, respectively. We find that over the historical period, nitrogen deposition (14%) and carbon‐nitrogen synergy (14%) were significant contributors to the current terrestrial carbon sink, suggesting that long‐term increases in nitrogen deposition led to a substantial increase in CO_2_ fertilization. However, relative to the contemporary baseline, changes in nitrogen deposition and carbon‐nitrogen synergy had no substantial contribution to the 21st century increase in global carbon uptake. Nonetheless, we find that increased nitrogen deposition in East Asia since the early 1990s contributed 50% to the overall increase in net biome production over this region, highlighting the importance of carbon‐nitrogen interactions. Therefore, potential large‐scale changes in nitrogen deposition could have a significant impact on terrestrial carbon cycling and future climate.

## Key Points

- Increased CO_2_ concentrations and nitrogen deposition combined synergistically to increase the land carbon sink over the 20th century
- Carbon‐nitrogen synergies do not appear responsible for the increase in land carbon sink since the beginning of the 21st century

## 1 Introduction

Fossil fuel CO_2_ emissions have rapidly increased since the turn of this century, at rates almost doubling those of the previous three decades (Hansen et al., 2013). Annual growth rates of atmospheric CO_2_ have been, somewhat surprisingly, relatively low during this period, which may imply that the global carbon sink has considerably strengthened (Ballantyne et al., 2012; Keenan et al., 2016). During the same epoch, there is also evidence of a decrease in land use and land cover change (LULCC) emissions (Houghton et al., 2012; Le Quéré et al., 2018) and a parallel increase in the strength of the ocean carbon sink (DeVries et al., 2017; Le Quéré et al., 2018; Rödenbeck et al., 2014), but both of these trends appear insufficient to account for the low growth of atmospheric CO_2_ (Le Quéré et al., 2018). As a result, the terrestrial carbon sink (estimated as the *residual* in the global carbon budget; Le Quéré et al., 2016) does exhibit a sharp increase since the early 2000s. An increasing terrestrial carbon sink since the early 2000s is also consistent with independent lines of evidence based on forest inventories (Pan et al., 2011) and process‐based modeling studies (e.g., Le Quéré et al. (2018)).

Various observation‐based (Clark et al., 2013; Los, 2013; Norby et al., 2005; Terrer et al., 2016) and modeling (Cheng et al., 2017; Keenan et al., 2016; Schimel et al., 2015; Sitch et al., 2015; Zhu et al., 2016) studies have highlighted the role elevated CO_2_ levels have on photosynthesis and water‐use efficiency in explaining the increase in the terrestrial carbon sink over recent decades. Schimel et al. (2015) estimated that 60% of the contemporary (1990–2007) terrestrial sink is due to increased atmospheric CO_2_ concentrations. However, if increased plant carbon uptake via the CO_2_ fertilization effect alone was the main driver behind the increase in global net carbon uptake since the turn of this century, we would expect a more transient increase over time (in line with gradual changes in atmospheric CO_2_ concentrations) than what is observed. Further, the strength of the CO_2_ fertilization effect based on carbon cycle simulations has been disputed by others, arguing that models tend to overestimate this effect (Gerber et al., 2010, 2013; Hungate et al., 2003; Huntzinger et al., 2017; Smith et al., 2016; Wieder et al., 2015) because they neglect important processes (e.g., role of colimitation by nutrients) that potentially limit the CO_2_ fertilization effect.

Nitrogen availability may constrain the response of ecosystem productivity to rising levels of atmospheric CO_2_ (Bonan & Levis, 2010; Churkina et al., 2009; Norby et al., 2010; Thornton et al., 2007; Zaehle, 2013; Zaehle & Dalmonech, 2011) via its role as an essential plant nutrient that constrains productivity globally (LeBauer & Treseder, 2008; Vitousek & Howarth, 1991). Nitrogen has been found to be particularly important for plant productivity in middle and high latitudes but may also be important in tropical regions (Elser et al., 2007; Fisher et al., 2012; LeBauer & Treseder, 2008). Any additional nitrogen added to the terrestrial biosphere could therefore enhance carbon storage directly by alleviating nitrogen limitation on plant productivity and indirectly by permitting a further plant response to CO_2_ fertilization (referred to as carbon‐nitrogen synergy). Synergistic effects can arise when high CO_2_ concentrations bring about nitrogen limitation, which is alleviated by concurrent rises in nitrogen deposition. Recent studies indicate enhanced terrestrial carbon uptake in the range of 0.2–0.5 Pg C/year (Churkina et al., 2009; Liu & Greaver, 2009; Wang et al., 2017; Zaehle, 2013) due to the direct effect of increased terrestrial nitrogen deposition from anthropogenic activities (~30 Tg N/year in 1850 to ~80 Tg N/year presently; Kanakidou et al., 2016). This enhancement of terrestrial carbon uptake would be equivalent to 10–20% of the total modern carbon sink. Since 1860, humans have doubled nitrogen inputs to the biosphere predominately through fossil fuel burning and agricultural intensification (Galloway et al., 2004; Gruber & Galloway, 2008). Over the last two decades the trends of nitrogen emissions and subsequent deposition have differed regionally. East Asian deposition has increased substantially (Jia et al., 2014, 2016; Liu et al., 2013), whereas European (Banzhaf et al., 2015; De Vries & Posch, 2011; Waldner et al., 2014) and North American (Du et al., 2014) nitrogen deposition is thought to have decreased during this time.

The influence of climate variability (via alterations in temperature, precipitation, cloudiness, and seasonal pattern) on changes in terrestrial carbon fluxes and storage may also be substantial (Ahlstrom et al., 2015; Baldocchi et al., 2016; Cox et al., 2013; Frank et al., 2015; Reichstein et al., 2013). The recent *warming hiatus* (1998–2013) was identified as a potential key mechanism behind the increased land sink during this period via reduced ecosystem respiration (Ballantyne et al., 2017). Hansen et al. (2013) speculated that the parallel increase in global fossil fuel emission and the land carbon sink since the turn of this century may be a result of carbon uptake mechanisms that are controlled by the emissions themselves, namely a larger fraction of diffuse solar radiation from increased sulfate aerosol emissions and increased nitrogen deposition. However, the *diffuse radiation* mechanism has been shown to play only a minor role at global scale (O'Sullivan et al., 2016). Alternatively, the concurrent rise in both anthropogenic carbon and nitrogen emissions (predominantly from East Asia) could have worked in tandem to fertilize the terrestrial biosphere via a combination of direct fertilization by increased nitrogen deposition along with an increased CO_2_ fertilization effect due to alleviation of nitrogen limitation. The latter synergistic effect can be observed in ecosystems colimited by various factors, which when relieved together lead to a strong synergistic response (De Vries et al., 2009, 2014; Finzi et al., 2007).

In this study, we investigated the hypothesis that the parallel increases in fossil fuel emissions and the land carbon sink since the turn of this century are causally linked through the individual and synergistic effects of increased atmospheric CO_2_ concentrations and nitrogen deposition on ecosystem productivity. We used a process‐based model of the terrestrial biosphere with fully interactive carbon‐nitrogen cycling. We analyzed sets of factorial simulations for two different periods (1900–2016 and 1990–2016) in order to quantify the effects of various model drivers (CO_2_, nitrogen, and climate) relative to historical and contemporary baselines.

## 2 Methodology

### 2.1 Model Description

For this study, we used the Community Land Model version 4.5 (CLM4.5‐BGC), which simulates biophysical, hydrological, and biogeochemical exchange processes between the land and the atmosphere (Oleson et al., 2013). CLM4.5‐BGC is fully prognostic with regards to carbon and nitrogen state variables in the vegetation, litter, and soil organic matter pools. The model also prognostically simulates the seasonal cycle of vegetation growth/decay, leaf area index, and vegetation height and includes explicit parameterizations of fire and harvest disturbance processes. We use a version of the model that includes an improved biogeochemistry scheme (CLM4.5‐BGC; Thornton et al., 2007). Compared to previous versions of the model, these improvements include vertically resolved carbon and nitrogen soil dynamics, a new decomposition scheme, and a more detailed representation of nitrification and denitrification (Koven et al., 2013). As a result of these updates, simulated fluxes and pools (of carbon and nitrogen) more closely reflect observational data (Koven et al., 2013). Also 20th century carbon dynamics are more realistic due to higher terrestrial carbon uptake because of reduced nitrogen constraints and longer turnover times for decomposing carbon (Koven et al., 2015).

While CLM4.5‐BGC has been described in detail (Oleson et al., 2013), we briefly summarize some key processes that are of relevance for this study. In CLM4.5‐BGC, leaf stomatal conductance (*g*_s_) is coupled to photosynthesis based on the Ball‐Berry model (Collatz et al., 1991; Sellers et al., 1996):

(1) $$\;g_{s}=m\frac{A_{n}}{c_{s}/P_{\mathrm{atm}}}h_{s}+b\beta_{t},$$

where *A*_n_ is the leaf photosynthesis rate, *c*_s_ is the CO_2_ partial pressure at the leaf surface, *P*_atm_ is the atmospheric pressure, *h*_s_ is the relative humidity at the leaf surface, *m* is a plant functional type (PFT) specific slope coefficient, and *b* is a PFT specific minimum stomatal conductance, regulated by the soil moisture stress factor *β*_t._ *β*_t_ranges between 0 (maximum water stress) and 1 (minimum water stress) and works to reduce the minimum stomatal conductance *b*. Further, *β*_t_ impacts *g*_s_ through its influence on *A*_n_, by scaling the maximum rate of carboxylation (*β*_t_*V*_cmax_).

Additionally, *A*_n_ depends (in part) upon the internal leaf CO_2_ partial pressure (*c*_i_) via Fick's law as follows:

(2) $$c_{i}=c_{a}-\left(1.4r_{b}+1.6r_{s}\right)P_{\mathrm{atm}}A_{n},$$

where *c*_a_ is the atmospheric CO_2_ partial pressure, *r*_b_ is the leaf boundary layer resistance, and *r*_s_ is the stomatal resistance. The equations for *c*_i_, *g*_s_, and *A*_n_ (not shown) are solved iteratively until *c*_i_ converges. This formulation couples the carbon and water cycles and both photosynthesis and stomatal conductance are reduced in dry conditions. Both *g*_s_ and *A*_n_ are solved separately for sunlit and shaded conditions and scaled through the canopy (as a function of leaf area index) to determine canopy level conductance and potential GPP (GPP_pot_).

Nitrogen limitation is modeled through downscaling GPP_pot_ depending on available nitrogen and required nitrogen by new carbon growth (Oleson et al., 2013; Thornton et al., 2007). Actual GPP is defined as follows:

(3) $$\mathrm{GPP}={\mathrm{GPP}}_{\mathrm{pot}}\left(1-f\right),$$

with the nitrogen scaling factor (*f*) defined as follows:

(4) $$f=\frac{{\mathrm{CF}}_{\text{avail}\_\text{alloc}}-{\mathrm{CF}}_{\text{alloc}}}{{\mathrm{GPP}}_{\mathrm{pot}}},$$

where CF_avail_alloc_ is the carbon flux from photosynthesis available for new growth, after accounting for maintenance respiration, and CF_alloc_ is the carbon allocation to new growth. In a first step, plant nitrogen demand is calculated depending on the amount of carbon available for growth (CF_avail_alloc_) and fixed C:N stoichiometry for each part of the vegetation (leaves, roots, and wood) for each PFT on the soil column. The plant demand for nitrogen is (partially) compensated for by translocating nitrogen from senescing leaves. Total plant nitrogen demand is reduced by this translocating flux to give the plant demand for mineral nitrogen from the soil. The combined nitrogen demand for all PFTs and heterotrophic demand from the soil (immobilization) compete for available soil nitrogen. Plant nitrogen uptake is then calculated (depending on the ratio of demand to supply), which is then used along with allometric relationships and C:N stoichiometry to calculate CF_alloc_.

Soil decomposition rates are also influenced by nitrogen availability. For decomposition from each upstream to downstream pool, a nitrogen source/sink term is calculated depending on the carbon and nitrogen content of each pool. Therefore, depending on plant demand for soil nitrogen, decomposition fluxes can be downregulated if nitrogen supply is limited. In addition to the rapid cycling of nitrogen in the plant‐litter‐soil system, CLM4.5 simulates dynamics of the *external* nitrogen cycle, with inputs of bioavailable nitrogen entering the terrestrial ecosystem through biological fixation and atmospheric deposition. Nitrogen leaves the system through losses due to fire, denitrification, and leaching. Additions from deposition and biological fixation are added straight to the mineral NH_4_^+^ pool, where plants and microbes compete for the nitrogen. This representation of carbon‐nitrogen interactions in CLM4.5‐BGC leads to a strong coupling between heterotrophic respiration and plant productivity, as respiration depends on organic matter produced and productivity depends on the nutrients made available through the decomposition of this organic matter (Thornton et al., 2007). Hence, further to the positive impact nitrogen deposition has upon plant productivity, another important pathway for nitrogen to fertilize plant growth is through warminginduced increases in nitrogen mineralization, a process which is also simulated in CLM4.5‐BGC (Thornton et al., 2007).

The model driver data used include nitrogen deposition for the period 1850–2000 from simulations based on the Community Atmosphere Model version 3.5 using historical nitrogen emissions (Lamarque et al., 2010). For the more recent period 2000–2016, we use nitrogen deposition fields generated following the emissions from Representative Concentration Pathway 8.5 (also using the Community Atmosphere Model version 3.5; Lamarque et al., 2011), as this most closely matches current emission levels (Peters et al., 2013). Due to the temporal averaging of emissions data (linear interpolation between decadal means), there is a smooth transition (no step changes between years) between emission inventories at the year 2000 and hence the deposition fields used.

Climate driver data used stem from the Climatic Research Unit ‐ National Centers for Environmental Prediction (version 7) data set (Viovy, 2018) (0.5° spatial and 6‐h temporal resolution), which is designed to drive CLM over long timer periods and aggregated/interpolated to the CLM4.5 spatial resolutions of 1.25° × 0.9375° and 30‐min time step. CRUNCEP is a combination of two data sets: CRU TS3.2 0.5° × 0.5° monthly data over the period 1901–2002 (Harris et al., 2014) and the NCEP reanalysis 2.5° × 2.5° six‐hourly data covering 1948–2016 (Kalnay et al., 1996). Further, we also used prescribed annual, globally averaged CO_2_ concentrations from the Earth Systems Research Laboratory (Dlugokencky & Tans, 2017). We used fixed present‐day land cover as described in section 21.3.3 of Oleson et al. (2013), meaning we did not consider land‐use and land‐cover change in this study since our focus was broadly on carbon‐nitrogen interactions.

### 2.2 Model Experiments

#### 2.2.1 The Extended Period 1901–2016

We performed a set of factorial simulations to assess the land carbon cycle response to increasing atmospheric CO_2_, nitrogen deposition, and climate changes, as well as the interactions between these drivers. This design allowed estimation of the effects of individual drivers on carbon pools and fluxes and hence on the overall terrestrial carbon budget during the period 1901–2016. Global annual means of model drivers (climate, nitrogen deposition, and atmospheric CO_2_) are shown in Figure S1 (in the supporting information), and the spatial distribution of changes over this period is shown in Figure S2. Our model spin‐up procedure followed that of the multimodel TRENDY study (Sitch et al., 2015) to be able to compare the results of this study to the TRENDY ensembles. It entails cycling early 20th century climate (1901–1920) with atmospheric CO_2_ concentrations and nitrogen deposition of the year 1860 until carbon pools and fluxes were in a steady state. The model then ran from 1861 to 1900 with varying CO_2_ and nitrogen deposition and the same climate cycles as in the first step. We then ran a set of factorial offline experiments over 1901–2016 with varying CO_2_, climate, nitrogen deposition, and fixed present‐day land use (see Table 1).

**Table 1:** Summary of Factorial Model Simulations With CLM4.5‐BGC

Experiment	CO_2_	Nitrogen deposition	Climate
S1	C	C	C
S2	T	C	C
S3	C	T	C
S4	C	C	T
S5	T	T	C
S6	T	C	T
S7	C	T	T
S8	T	T	T

*Note*. C (constant) indicates that 1900 values are used for atmospheric CO_2_ and nitrogen deposition and that 1901–1920 climate is recycled. T (transient) indicates that historically varying CO_2_, nitrogen deposition, and climate are used.

From this set of eight simulations, we estimated the contribution from each driver to changes in net primary production (NPP), heterotrophic respiration (RH), net biome production (NBP; estimated through NBP = NPP – RH − fire), and total ecosystem carbon as follows: CO_2_ fertilization = (S2 − S1), nitrogen deposition = (S3 − S1), climate (S4 − S1*), carbon‐nitrogen synergy = (S5 − S2) − (S3 − S1), carbon‐climate synergy = (S6 − S4) − (S2 − S1), and the combined effect = (S8 − S1*). Here simulation S1* represents the linear trend (from 1901 to 2016) in annual means of NPP, NBP, and total ecosystem carbon based on experiment S1. We use the trend in S1 rather than annual means to preserve the interannual variability of climate in the *Climate* and *Combined* contributions. Taking the difference between the simulations removes the background carbon trends from the nonequilibrium initial conditions (see Bonan and Levis, 2010).

We calculate the change in NPP and NBP due to each driver over the study period by differencing the 2007–2016 and 1901–1910 means. To statistically test for a difference between the two decades we use a Mann‐Whitney *U* test. As a result of our experiment design, contributions from CO_2_ fertilization, nitrogen deposition, carbon‐nitrogen synergy, and carbon‐climate synergy use early 20th century climate as a source of variance in both decades (1901–1910 and 2007–2016). For the contributions from climate, and *combined*, the *actual* climate variability in each decade is the source of variance.

#### 2.2.2 The Recent Period 1990–2016

We performed a second set of experiments for the more recent period (1990–2016) using initial conditions obtained from experiment S8 (at 1990) by branching out of experiment S8 (Table 1). Simulations are performed in a similar manner to the extended period; however, our constant values were from 1990 for atmospheric CO_2_, nitrogen deposition, and climate. We performed these simulations of the recent period to quantify recent changes in carbon/nitrogen cycling relative to a more contemporary baseline. Such an analysis would be more closely aligned with the time frame of our main aim of evaluating explanations for the terrestrial sink increase since the turn of this century. Contributions from each factor are calculated through factorial simulations, similar to the extended period.

### 2.3 Diagnosing Model Results

Nitrogen limitation (N‐lim) is a key metric in assessments of carbon‐nitrogen coupling and is directly estimated in CLM4.5‐BGC through the ratio of actual GPP to potential GPP (GPP that would occur without nitrogen limitation) at each time step and thus is a scalar between 0 and 1, with high/low N‐lim values indicating low/high nitrogen limitation.

In diagnosing our model results, we evaluate N‐lim along with the Γ factor that expresses the sensitivity of terrestrial carbon storage to atmospheric CO_2_. For the Γ factor, we adopt the definition: $\Gamma_{X}=\frac{{\mathrm{∆TEC}}_{X}}{{∆C}_{a}}=\frac{∆\left({\mathrm{TEC}}_{Y}-{\mathrm{TEC}}_{Z}\right)}{∆C_{a}}$, where *∆TEC*_*X*_ is the change in total ecosystem carbon (Pg C) due to factor *X* over a certain period, calculated as the difference between simulations *Y* and *Z* (see Table 2). *∆C*_a_ is the change in atmospheric CO_2_ (ppm) over the same period. For the extended period, we focus on the change in TEC and *C*_a_ from 1901–1910 to 2007–2016 and for the recent period on the change from 1990–1996 to 2010–2016.

**Table 2:** Summary of Simulations Used in the Calculations for Γ

$\Gamma_{{\mathrm{CO}}_{2}}$	$\Gamma_{{\mathrm{CO}}_{2}+\text{CNsyn}}$	$\Gamma_{{\mathrm{CO}}_{2}+\text{CNsyn}+\text{NDEP}}$
*∆*(TEC_S2_ − TEC_S1_)/*∆C*_a_	*∆*(TEC_S5_ − TEC_S3_)/*∆C*_a_	*∆*(TEC_S5_ − TEC_S1_)/*∆C*_a_

*Note*. Γ is calculated for the direct CO_2_ effect $\Gamma_{{\mathrm{CO}}_{2}}$, the direct CO_2_ and carbon‐nitrogen synergy effects $\Gamma_{{\mathrm{CO}}_{2}+\text{CNsyn}}$, and finally the direct CO_2_, direct nitrogen deposition effects, and the synergy between them $\Gamma_{{\mathrm{CO}}_{2}+\text{CNsyn}+\text{NDEP}}$.

## 3 Results

### 3.1 Long‐Term Changes in Net Terrestrial Carbon Uptake and Attribution of Underlying Drivers

To evaluate our hypothesis that nitrogen deposition, CO_2_ fertilization, and their interactions have enhanced the terrestrial carbon sink, we first analyze our CLM4.5‐BGC model simulations of the carbon component fluxes NPP and RH, as well as NBP covering the extended period over the last century.

**Figure 1:**
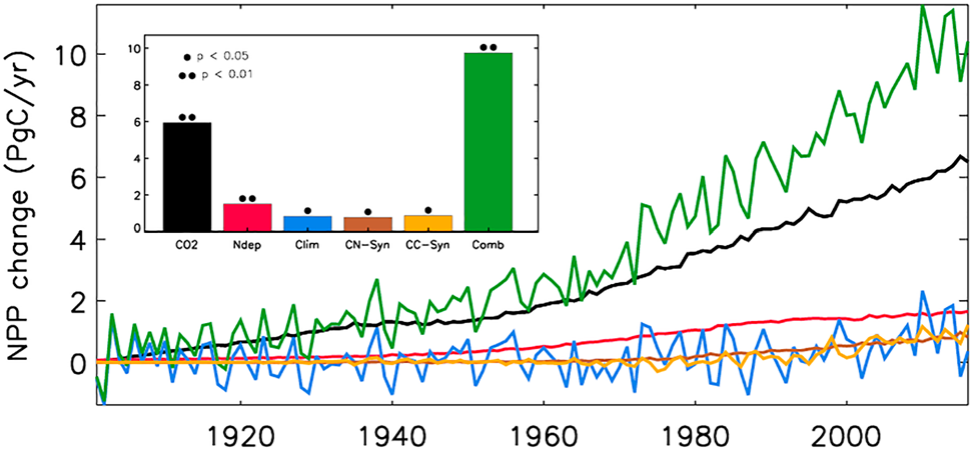
Global, annual mean change in net primary production (NPP; Pg C/year) relative to the control simulation (S1) during 1901–2016 due to CO_2_ fertilization (CO_2_), nitrogen deposition (Ndep), climate change (Clim), carbon‐nitrogen synergy (CN‐Syn), carbon‐climate synergy (CC‐Syn), and the combined effects (Comb). Inset shows the change in NPP from 1901–1910 to 2007–2016. Statistically significant (• *p* < 0.05, •• *p* < 0.01; Mann‐Whitney *U* test) changes are highlighted.

At the global scale, simulated NPP increased substantially over the 20th century to present day from 56.2 (mean of 1901–1910) to 66.0 Pg C/year (mean of 2007–2016) with positive contributions from all drivers considered, including rising CO_2_ concentrations (referred to as CO_2_ fertilization), nitrogen deposition, climate, and carbon‐nitrogen as well as carbon‐climate synergies (Figure 1 and Table 3). The relative contribution of these drivers to this overall NPP increase amounts to 60% for increased CO_2_, 15% for nitrogen deposition, 8% for carbon‐nitrogen synergy, 9% for carbon‐climate synergy, and 8% for climate. Both CO_2_ fertilization and nitrogen deposition individually caused a smooth, transient increase in NPP, in line with the trajectory of the corresponding drivers (see Figure 1 and Figure S1). The positive carbon‐nitrogen synergistic contribution to NPP implies that (as expected) the efficiency of the CO_2_ fertilization effect is enhanced as nitrogen limitation is diminished (through nitrogen deposition). In addition, a similar positive contribution of comparable magnitude is observed for the carbon‐climate synergistic effect.

**Table 3:** Change in Global NPP and NBP (Pg C/year) for the Extended ([2007–2016]–[1901–1910]) and Recent ([2010–2016]–[1990–1996]) Periods

Period	Variable	Change due to each driver (Pg C/year)						
		CO_2_	NDEP	Climate	CN‐SYN	CC‐SYN	Sum of three effects (% of Combined)	Combined
Extended	NPP	5.93	1.50	0.84	0.77	0.86	8.27 (85%)	9.75
	NBP	2.39	0.34	−1.07	0.35	0.44	1.66 (72%)	2.31
Recent	NPP	1.91	0.03	1.22	0.01	0.24	3.16 (93%)	3.41
	NBP	1.22	0.03	−1.17	0.00	0.18	0.08 (30%)	0.27

*Note*. Positive values for NBP indicate a sink of carbon to the land surface. SUM of three effects indicates the sum of CO_2_, NDEP, and Climate. NPP = net primary production, NBP = net biome production.

**Figure 2:**
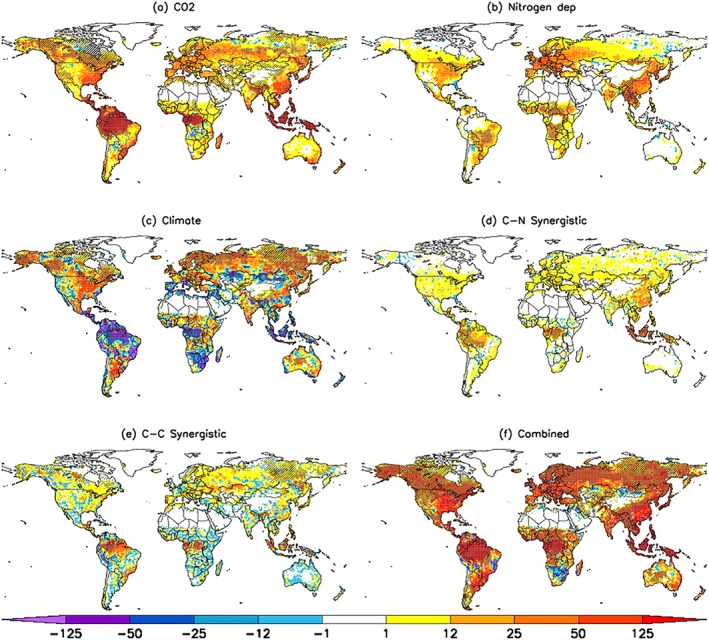
Spatial patterns of net primary production (NPP) change (g C/m^2^/year) as a result of all drivers considered. Maps show single driver contribution from (a) CO_2_ fertilization, (b) nitrogen deposition, (c) climate, (d) CN‐synergy, (e) CC‐synergy, and (f) the combined effect, respectively. The patterns are based on a set of factorial simulations (see section 2). NPP changes shown here are calculated as the difference between 2007–2016 (final decade) and 1901–1910 (first decade) mean values. Significant (*p* < 0.05; Mann‐Whitney *U* test) changes highlighted with hatching.

A spatially explicit analysis of the factorial simulations shows that the CO_2_ fertilization effect is most profound in tropical regions (Figure 2a). Substantial contributions from nitrogen deposition are also evident over the industrialized regions of Europe, East Asia, and North America and the agricultural regions of India and Southeast Asia (Figure 2b). While NPP increases due to nitrogen deposition have the largest footprint in industrialized regions, the associated NPP response also depends on the nitrogen limitation of a given ecosystem. This is apparent in the grasslands of Africa and South America, where nitrogen deposition (Figure S2) induced a substantial NPP response (Figure 2b). The increase in NPP due to climate can be attributed to middle and high northern latitudes, where warming has led to a longer growing season and increased soil moisture (Figure S3), enhancing annual net plant carbon uptake (Figure 2c). Further, warming enhanced nitrogen mineralization in these soils (Figure S4) increasing plant productivity, which is also captured in the climate response. The positive carbon‐nitrogen synergistic contribution is prevalent in tropical forests and East Asia (Figure 2d), regions that are also exhibiting high sensitivity to CO_2_ fertilization (Figure 2a). Similarly, positive carbon‐climate synergistic effects are substantial in the tropics, as well as regions in the middle/high latitudes (Figure 2e).

**Figure 3:**
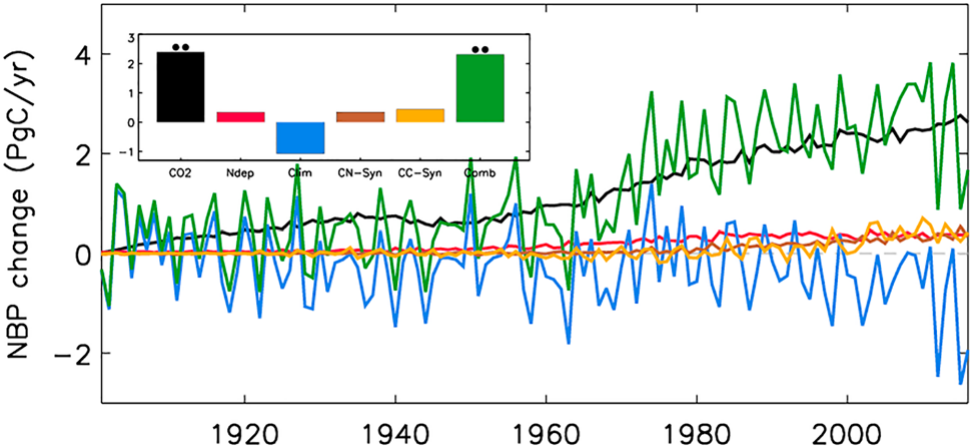
Global, annual mean change in net biome production (Pg C/year) relative to the control simulation (S1) during 1901–2016 due to CO_2_ fertilization (CO_2_), nitrogen deposition (Ndep), climate change (Clim), the combined effect (Comb), carbon‐nitrogen synergy (CN‐Syn), and carbon‐climate synergy (CC_Syn). Zero line is shown in gray. Inset is the change in net primary production from (1901–1910) to (2007–2016). Statistically significant (• *p* < 0.05, •• *p* < 0.01; Mann‐Whitney *U* test) changes are highlighted.

Globally, NBP has increased from 0.8 to 3.2 Pg C/year (1901–1910 to 2007–2016 means) with positive contributions from CO_2_ fertilization, nitrogen deposition, carbon‐nitrogen synergy, and carbon‐climate synergy while an overall negative contribution from climate (Figure 3, Table 3). The relative contribution of these drivers to this overall NBP increase amounts to 99% for increased CO_2_, 14% for nitrogen deposition, 14% for carbon‐nitrogen synergy, 18% for carbon‐climate synergy, and −45% for climate. While the CO_2_ fertilization effect steadily contributed to NBP changes throughout the whole period, nitrogen deposition induced NBP increases became significant from the 1970s onward (Figure 3), a period of increased anthropogenic nitrogen deposition (fossil fuel NO_*x*_ and agricultural NH_*x*_; Lamarque et al., 2010). Results also show that the carbon‐nitrogen synergistic effect is as large as the effect from nitrogen deposition alone, implying that additional nitrogen had a large positive impact on CO_2_ fertilization. NBP is highly sensitive to climatic changes especially at interannual timescales (Figures 3 and S1). Overall, changes in climate have led to a net carbon source, with accelerated losses since the 1990s, due to warming‐induced soil respiration rates increasing faster than NPP.

**Figure 4:**
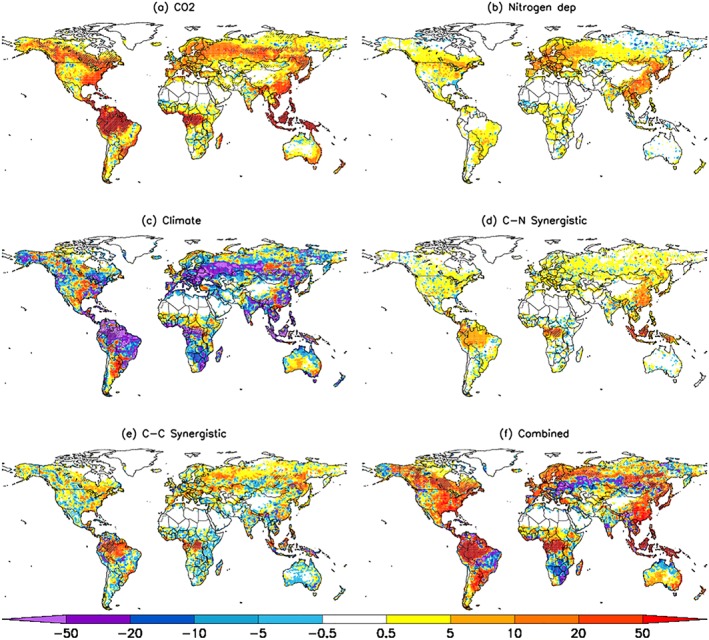
Spatial patterns of net biome production change (g C/m^2^/year) due to (a) CO_2_ fertilization, (b) nitrogen deposition, (c) climate, (d) CN‐synergy, (e) CC‐synergy, and (f) combined effect. The synergistic effect is calculated as the difference between 2007–2016 and 1901–1910 mean values. Significant (*p* < 0.05; Mann‐Whitney *U* test) changes highlighted with hatching.

The spatial distribution of changes in NBP over roughly the last century from CO_2_ fertilization and nitrogen deposition mirrors those seen in NPP (Figures 4a, 4b, 2a, and 2b) and is consistent with the notion of a strong influence of NPP on net carbon uptake. Interestingly, increased nitrogen deposition seems to have had no direct effect on tropical forest net carbon uptake (but instead increased the efficiency of CO_2_ fertilization: the CN synergistic effect as discussed below). NBP decreases due to climate can be attributed predominately to tropical regions as well as large areas across Eurasia and North America (Figure 4c). Both the carbon‐nitrogen and carbon‐climate synergistic effects follow the NPP patterns of large tropical increases, as well as vast areas of the middle to high latitudes (Figures 4d and 4e). These synergistic effects occur when there is both a high sensitivity to CO_2_ fertilization and a concurrent release of nitrogen limitation. This is seen in the case of tropical forests where increased atmospheric CO_2_ concentrations increases nitrogen limitation that is then alleviated with simultaneous increases in nitrogen deposition (Figure 4d). Overall, the majority of the vegetated land surface has increased net carbon uptake over the historical period, with the tropics, East Asia, North America, and northern Eurasia dominating (Figure 4f). However, areas in South America, Southern Africa, and the Eurasian Steppe are now carbon sources to the atmosphere.

**Figure 5:**
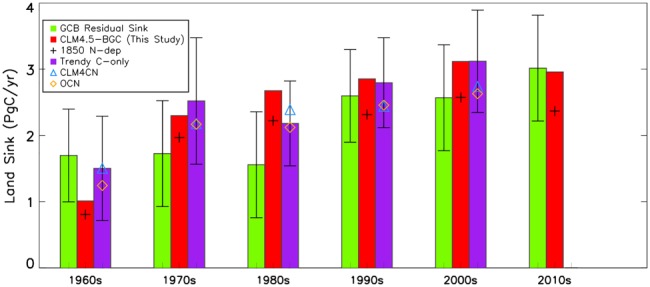
Multiple estimates of the decadal mean land carbon sink for the 1960s–2010s based on the GCB, TRENDY (S2), and this study. Carbon sink (Pg C/year) estimates correspond to the GCB residual (green bars), this study (red bars), this study with 1900 nitrogen deposition (black crosses), and the ensemble‐mean from Trendy based on carbon‐only models (purple bars). Estimates for the two Trendy models with interactive nitrogen are also shown: CLM4CN (blue triangles) and OCN (orange diamonds). The GCB error bars represent the uncertainty in the corresponding sink estimate as provided by Le Quéré et al. (2016). The error bars for the Trendy estimate represent one standard deviation based on the multimodel ensemble mean. The final decade (2010s) captures the mean land sink for the period 2010–2016. For our study, the influence of anthropogenic nitrogen deposition (direct and synergistic effects) can be inferred by the difference between the black cross and red bar. This effect has grown from 0.2 Pg C/year in 1960s to 0.7 Pg C/year in 2010s. Note that the TRENDY simulations and the model results from this study do not consider LULCC, and the GCB residual sink inherently accounts for LULCC fluxes (see also section 2).

Recent published findings based on the Global Carbon Budget (GCB) show that the land carbon sink has increased over the last five decades (Le Quéré et al., 2018). In the GCB, the land sink is estimated as the residual in the global carbon mass balance between fossil fuel and land‐use emissions, atmospheric CO_2_ growth rates, and ocean uptake. This residual sink has increased from ~1.5 to ~3 Pg C/year from the 1960s to the 2000s (Figure 5). The estimated decadal carbon sinks in our study are in general agreement and within the uncertainties of the GCB estimates, giving some confidence in our modeled magnitude (Figure 5).

We also compared our results to those based on the recent TRENDY multimodel intercomparison, which consider the influence of varying atmospheric CO_2_ concentrations and climate on carbon fluxes (S2 simulations; Sitch et al., 2015). As carbon‐nitrogen interactions are a focal point of this study, we compared our results with the TRENDY models that include a coupled carbon‐nitrogen scheme (CLM4‐CN and OCN) separately to the *carbon‐only* models. While CLM4‐CN and OCN tend to simulate lower net carbon uptake compared to the carbon‐only mean, they are both still within the range spanned by the TRENDY carbon‐only ensemble (Figure 5). Therefore, while introducing a coupled carbon‐nitrogen scheme tends to have a strong influence on land carbon uptake in DGVMs (Friedlingstein & Prentice, 2010; Thornton et al., 2007; Zaehle et al., 2010), the structural difference between models is a larger source of uncertainty. Our results match the mean TRENDY carbon sink estimate well on decadal scales, albeit with noticeable differences in the 1960s and 1980s.

### 3.2 Recent Changes in Net Terrestrial Carbon Uptake and Attribution of Underlying Drivers

We next evaluated whether the marked strengthening of the terrestrial carbon sink since the turn of this century was due to the hypothesized causal link between concurrent changes in the sink and anthropogenic fossil fuel emissions (Hansen et al., 2013; Keenan et al., 2016). In a first step, we tested to what extent the model (with all drivers varied) captures the uptick in the residual land carbon sink since the turn of the century. The trend in the residual sink increased by 0.33 Pg C/year^2^ between 1990–2002 and 2002–2014 (Figure S5). Our model simulated a smaller increase of 0.08 Pg C/year^2^, in line with the change simulated by the TRENDY multimodel mean, 0.09 Pg C/year^2^ (Figure S5).

**Figure 6:**
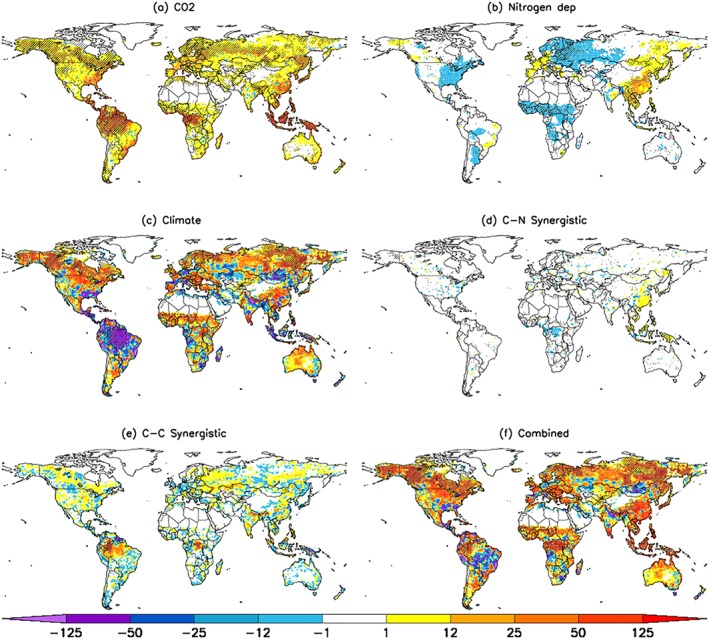
Spatial patterns of net primary production (NPP) change (g C/m^2^/year) due to (a) CO_2_ fertilization, (b) nitrogen deposition, (c) climate, (d) CN‐synergy, (e) CC‐synergy, and (f) combined effect. The synergistic effect is calculated as the difference between the combined effect and sum of effects. NPP changes shown here are calculated as the difference between 2010–2016 and 1990–1996 mean values. Significant (*p* < 0.05; Mann‐Whitney *U* test) changes highlighted with hatching.

Next, we performed factorial simulations starting from contemporary 1990 baseline conditions (see section 2) to attribute drivers and processes in the context of the more recent uptick in the terrestrial carbon sink. Corresponding results show a global NPP increase of 3.4 Pg C/year between the early 1990s (mean of 1990–1996) and the end of our study period (2010–2016), with CO_2_ fertilization and climate being the dominant drivers, accounting for 56% and 35% of the overall change, respectively (Table 3). On a global scale, terrestrial nitrogen deposition increased by 3% over this period (70 to 72 Tg N/year) and hence had little impact on NPP changes (Table 3). However, the effect of deposition differed across regions, reflecting the spatial pattern of nitrogen deposition. East Asia and Western Europe experienced enhanced NPP consistent with the positive change in nitrogen deposition over this period, whilst Eastern Europe, North America, and the African savannah had a decline in NPP consistent with a decrease in nitrogen deposition (Figures 6b and S6; Table S1). Northern Hemisphere warming between the two focal epochs (1990–1996 and 2010–2016) led to widespread NPP increases in the boreal regions of Eurasia and North America (Figures 6c and S7). In regions outside of the northern high latitudes, changes in water availability (e.g., soil moisture) also drove positive NPP changes (Figure S8). In contrast to the extended study period (1900–2016, see above), the carbon‐nitrogen synergistic effect is near zero over the recent period, with only a small increase in East Asia (Figure 6d). Conversely, carbon‐climate synergy has a small but noticeable positive contribution to NPP (Figure 6e). In this case, tropical forests in South America and Central Africa have a positive response, whilst most other regions exhibit small and spatially heterogeneous responses. Potential factors underlying the difference in simulated synergistic effects between the extended and the more recent periods are discussed below.

At global scale, changes in climate led to a loss of NBP by 1.17 Pg C/year between the two focal periods 1990–1996 and 2010–2016, whereas CO_2_ fertilization increased NBP by 1.22 Pg C/year (Table 3). Changes in nitrogen deposition played only a minor role, sequestering an additional 0.03 Pg C/year, whilst carbon‐nitrogen synergy had an insignificant contribution. Carbon‐climate synergy effects induced a relatively small (but important) positive increase in NBP of 0.18 Pg C/year, which in combination with all other drivers considered led to an overall increase of 0.27 Pg C/year between 1990–1996 and 2010–2016, with CO_2_ fertilization dominating the response (Table 3).

**Figure 7:**
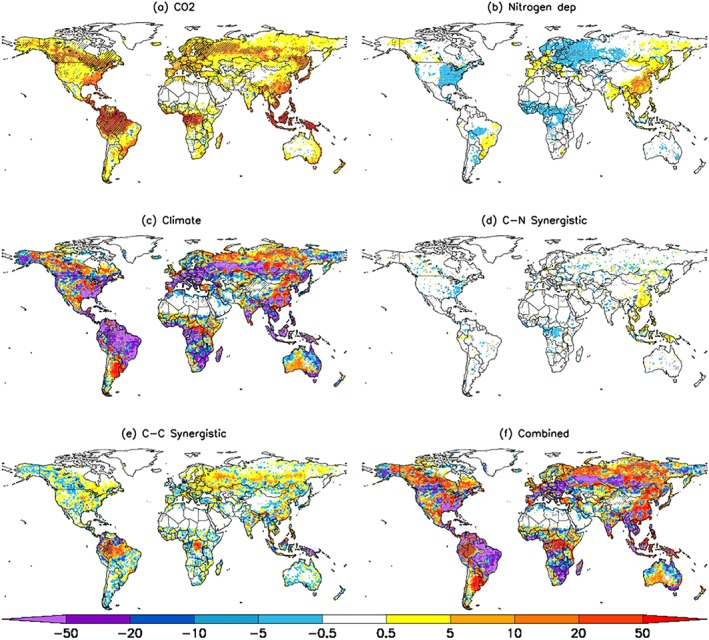
Spatial patterns of net biome production (NBP) change (g C/m^2^/year) due to (a) CO_2_ fertilization, (b) nitrogen deposition, (c) climate, (d) CN‐synergy, (e) CC‐synergy, and (f) the combined effect. The synergistic effect is calculated as the difference between the combined effect and sum of effects. NBP changes shown here are calculated as the difference between 2010–2016 and 1990–1996 mean values. Significant (*p* < 0.05; Mann‐Whitney *U* test) changes highlighted with hatching.

The spatial pattern of changes in NBP between 1990–1996 and 2010–2016 due to CO_2_ fertilization, nitrogen deposition, and CN‐synergy effects (Figures 7a, 7b, and 7d) were similar to the associated NPP pattern (see Figure 6) as expected since these drivers predominantly influence NBP through their effect on plant carbon uptake. Conversely, climate variations caused widespread declines in NBP due to either a combination of reduced NPP and increased soil respiration (such as in the Amazon) or respiration increases being larger than NPP increases, as observed over the middle/high latitudes (Figures 7c and S9). Carbon‐climate interactions led to significant increases in tropical forests and the forests of North America, Eurasia, and China (Figure 7e). The overall pattern of NBP change is dominated (on grid‐box scale) by climate variability, although CO_2_ fertilization effects are visible across the tropics (Figures 7c and 7f).

### 3.3 Sensitivity of Carbon‐Nitrogen Interactions for the More Recent and Extended Study Periods

We next examined the extent to which additional nitrogen deposition has changed the efficiency of the CO_2_ fertilization effect, in order to evaluate our hypothesis that nitrogen deposition has interacted with CO_2_ to increase the terrestrial carbon sink. Hence, in a next step, we considered the sensitivity of carbon storage to atmospheric CO_2_ concentrations (Γ = $\frac{\mathrm{∆TEC}}{∆C_{a}}$) and how this is modulated by carbon‐nitrogen interactions (see section 2). For the extended period covering roughly the last century, we find $\Gamma_{{\mathrm{CO}}_{2}}=1.32$ Pg C/ppm CO_2_ (Table 4). Including the carbon‐nitrogen synergistic contribution increased Γ by 0.08 Pg C/ppm CO_2_, and direct nitrogen deposition increased Γ by a further 0.21 Pg C/ppm. These estimates indicate that additional nitrogen enabled higher plant carbon uptake via more effective CO_2_ fertilization and that the direct effects from additional nitrogen were approximately twice that of the synergistic contribution (increase of 0.21 Pg C/ppm compared to 0.08 Pg C/ppm; Table 4).

**Table 4:** Γ Values (Pg C/ppm) for the Extended (1901–1910 to 2007–2016) and Recent (1990–1996 to 2010–2016) Periods

Period	$\Gamma_{{\mathrm{CO}}_{2}}$	$\Gamma_{{\mathrm{CO}}_{2}+\text{CNsyn}}$	$\Gamma_{{\mathrm{CO}}_{2}+\text{CNsyn}+\text{NDEP}}$
Extended	1.32	1.40	1.61
Recent	0.45	0.45	0.45

*Note*. Estimates are provided for Γ factors associated with the direct CO_2_ effect (CO_2_), CO_2_ and CN‐synergistic effects (CO_2_ + CNsyn), and the combined CO_2_ and nitrogen deposition effects, including CN‐synergy (CO_2_ + CNsyn + NDEP).

The magnitude of Γ and the impact of nitrogen are sensitive to the baseline of simulations because of the influence of background carbon trends and the current state of carbon and nitrogen pools. Therefore, we also focus on the recent period (1990–2016), enabling us to quantify the contribution from changes in nitrogen deposition to CO_2_ fertilization relative to a more contemporary baseline. Our method of calculating Γ removes background trends in total ecosystem carbon from nonequilibrium conditions (see section 2) and so purely captures the response to rising CO_2_ and nitrogen deposition relative to the chosen baseline. For the recent period (1990–2016), the removal of a background trend in total ecosystem carbon and the relatively large *∆C*_a_ since 1990 led to much lower Γ values of 0.45 Pg C/ppm (Table 4). The impact of direct nitrogen deposition on Γ is limited due to opposing regional impacts (increases in Western Europe and China and decreases in North America, Eastern Eurasia, and Africa) leading to an insignificant global effect (Table 4; Figures S6 and S10f). Furthermore, the rate of nitrogen deposition globally changed little between the two periods (1990–1996 to 2010–2016), and the small spatial extent of increased deposition (restricted to Western Europe and East Asia; Figure S6a) also limited the global synergistic response. Although Γ increases in China due to carbon‐nitrogen synergy, there is no response in Western Europe (Figure S10e). This lack of a synergistic effect is possibly due to the relatively low increase (7%) in deposition in Western Europe over this period, compared to the larger increase observed in East Asia (27%) (Table S1).

Overall, nitrogen deposition and associated synergistic effects have increased the sensitivity of the biosphere to atmospheric CO_2_ over the 20th century. However, relative to a modern baseline (which approximates the real‐world situation in regard to attributing mechanisms of the accelerated 21st century sink more closely), there are no synergistic effects.

### 3.4 Tracking Nitrogen Limitation for the Extended and More Recent Study Periods

**Figure 8:**
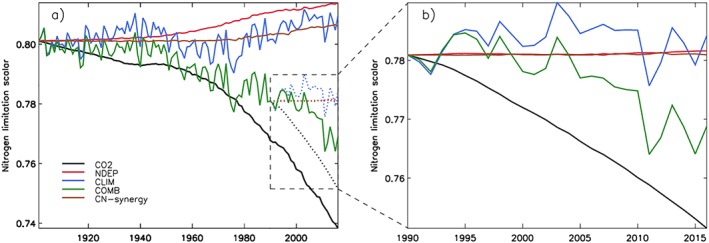
Changes in the global N limitation scalar for (a) extended simulations (1901–2016) and (b) short simulations (1990–2016). Changes are relative to a control simulation with no variables changing. Also note that the short simulations are relative to 1990 baseline. Contributions from CO_2_ (black), nitrogen deposition (red), climate (blue), combined (green), and CN‐synergy (brown) are shown. Note that the nitrogen limitation scalar represents the ratio of actual GPP to potential GPP (GPP in nitrogen saturated conditions), and so a decrease indicates higher nitrogen limitation.

We also evaluated how nitrogen availability regulates carbon uptake using a *nitrogen limitation scalar* (N‐lim), the ratio of actual GPP to *potential GPP* (simulated GPP before nitrogen limitation is imposed, see section 2). Globally, at the baseline of our extended study period (1901), nitrogen limitation reduced GPP by 20% (161.3 to 129.3 Pg C/year). At global scale, our trajectories of N‐lim showed decreases (increasing limitation) under increasing atmospheric CO_2_ (Figure 8a). Increased nitrogen deposition reduced the limitation, in line with expectations (Figure 8a). However, N‐lim is also sensitive to changes in climate, exhibiting large interannual variability with a positive trend (reduced limitation) from 1980s onward. Climate has a complex relationship with N‐lim due to the influence on both the amount of required nitrogen for climate‐driven changes in photosynthesis and available nitrogen (through impact on nitrogen soil remineralization rates), meaning the exact cause of the simulated behavior is difficult to diagnose. Carbon‐nitrogen synergy (the interaction of rising CO_2_ concentrations and rising nitrogen deposition) reduced nitrogen limitation from 1980 onward (Figure 8a), matching the period in which we see a synergistic response in NPP and NBP (Figures 1 and 3). For the carbon‐nitrogen synergistic contribution to be significant, additional nitrogen deposition needs to alleviate the progressive nitrogen limitation brought about by rising CO_2_ concentrations. This synergistic contribution only occurs when additional nitrogen is required by vegetation due to rising CO_2_ concentrations (as additional carbon inputs increase immobilization of nitrogen by plants and microbes), which in some ecosystems takes years to develop. This can be seen in the Amazon where N‐lim is constant until the 1970s and then decreases (higher nitrogen limitation) due to rising CO_2_, inducing an increase (reduced nitrogen limitation) in N‐lim from carbon‐nitrogen synergy (Figure S11).

With a focus on the more recent study period (1990 baseline), increased CO_2_ concentrations increase nitrogen limitation at global scale similar to the extended period (Figure 8b). In addition, climate warming has increased nitrogen mineralization rates, reducing the limitation, albeit this is masked to some extent by substantial interannual variability (Figure 8b). Importantly, over this timescale nitrogen deposition and carbon‐nitrogen synergy had a near zero contribution to changes in N‐lim. This is partly because the change in global nitrogen deposition between 1990 and 2016 was relatively small (Figure S12). However, nitrogen deposition increases in East Asia and West Europe, and decreases in North America over this period had noticeable but opposing influences on global N‐lim (Figure S13). Furthermore, the direct influence of nitrogen deposition on N‐lim was noticeable (in Western Europe and East Asia); there was, however, virtually no change due to carbon‐nitrogen synergy (Figures S13b and S13c). Therefore, additional nitrogen deposition in these regions was insufficient to alleviate substantially the nitrogen limitation brought about by high CO_2_ concentrations. It is difficult to pinpoint the exact cause of our simulated responses, but as previously stated, this is potentially due to the short time period considered, limiting the time for synergistic effects to arise.

## 4 Discussion

Understanding the mechanisms behind the ongoing changes in the terrestrial carbon cycle is critical for reliably predicting how the Earth system may change into the future. Here we assessed the hypothesis that increases in CO_2_ concentrations and nitrogen deposition (both linked to increasing rates in fossil fuel burning) worked synergistically to increase the terrestrial carbon sink since the turn of this century using a modeling approach. Significant effects are found over the historical period (1901–2016; Figure 1); however, relative to a modern baseline (1990), we find that both nitrogen deposition and carbon‐nitrogen synergy had no substantial contribution to the increased land sink since the turn of this century (Figures 6 and 7; Le Quéré et al., 2018), likely because global nitrogen deposition changed little during this period. Importantly, however, there have been significant shifts in the spatial patterns of nitrogen deposition and subsequent impacts on the carbon sink since the 1990s (Figures 7 and S6). This highlights the pivotal role nitrogen availability has upon the local carbon cycle. Several studies suggest that vegetation productivity is limited by nitrogen (Fisher et al., 2012; Janssens et al., 2010), and enhanced nitrogen deposition, predominantly from anthropogenic fossil fuel burning, is thought to have contributed positively to the historical terrestrial carbon sink (Fleischer et al., 2015; Thornton et al., 2007; Wang et al., 2017; Zaehle et al., 2010). Our current (2010–2016) sink estimate of 0.36 Pg C/year (Table 3) is in agreement with these previous studies (0.2–0.5 Pg C/year). We found that nitrogen deposition induced effects start to occur toward the latter decades of the 20th century when the additional nitrogen worked to offset the increased nitrogen limitation brought about by increasing CO_2_ concentrations (Figures 1 and 3; Finzi et al., 2006).

Tropical ecosystems are considered not limited by nitrogen (Hedin et al., 2009) but can become nitrogen limited as atmospheric CO_2_ concentrations rise. In our simulations, this process begins in the 1980s, at which point the direct CO_2_ fertilization effect is reduced (Figure S11). This increased nitrogen limitation is also an indication of when synergistic effects can develop, because from this time any additional nitrogen deposition can alleviate this limitation. Our estimate (1990s mean) of the synergistic contribution to the terrestrial sink (0.32 Pg C/year) is similar to that of Zaehle et al. (2010; 0.4 Pg C/year) but smaller than the Churkina et al. (2009) estimate of 0.7 Pg C/year (Table 3). Differences between estimates are not surprising given the complex interactions between the carbon and nitrogen cycles, different model parameterizations, and the use of offline (Zaehle et al., 2010) or coupled (Churkina et al., 2009) model simulations.

However, most studies that look to quantify the influence of nitrogen deposition on the land carbon sink perform long‐term multidecadal/century‐scale simulations (Bala et al., 2013; Churkina et al., 2009; Devaraju et al., 2016; Thornton et al., 2007; Wang et al., 2017; Zaehle et al., 2010) and so quantify changes in carbon cycling over given historical periods. In these modeling studies (and including this one), interaction effects are shown to develop over the course of many decades and so highlight how the magnitude of simulated nonlinear effects depend on the timescale and baseline considered. In this regard, the scientific community has given less attention to process attribution behind the post‐2000 carbon sink. Our analysis indicates that for the most recent decade, changes in nitrogen deposition and corresponding effects on CO_2_ fertilization had no influence on global carbon uptake (Figure 3). Thus, from our long‐term historical simulations, we conclude that additional nitrogen deposition has increased the sink by ~0.7 Pg C/year, but recent regional changes in deposition have not altered the nitrogen induced global sink. This is primarily due to opposing responses from nitrogen deposition increases in East Asia and Western Europe and decreases in North America and Eastern Europe, respectively, resulting in a small overall impact globally (Figures 6 and 7). Furthermore, increased uptake from carbon‐nitrogen synergy only occurs in regions of increased deposition (Churkina et al., 2009; Zaehle et al., 2010); however, we find that in both East Asia and Western Europe, high nitrogen limitation brought about by high atmospheric CO_2_ concentrations in this period inhibits carbon uptake, a constraint which is not fully alleviated by the extra nitrogen deposited. Despite the fact that evidence points toward nitrogen deposition induced East Asian greening and carbon sink increases in the last three decades (Gu et al., 2015; Piao et al., 2012, 2015; Zhu et al., 2017), our results suggest that a significant contribution from additional nitrogen deposition to the enhanced global land sink since the turn of this century is unlikely.

Thus, what processes and mechanisms are behind the post‐2000 increase in the land carbon sink? First, it should be noted that our modeled land carbon sink does not fully capture the magnitude of the uptick seen in the residual land sink since ~2000 (Figures 5 and S5), exhibiting a more transient increase. While the limited ability of reproducing this uptick appears to be model specific (Le Quéré et al., 2016), this may indicate that the model used is inadequately capturing and/or missing key processes. Yet our analysis indicates that even in a scenario with high nitrogen limitation, CO_2_ fertilization is the main driver behind the increased sink, a result consistent with previous modeling studies (Keenan et al., 2016; Schimel et al., 2015; Sitch et al., 2015), with nitrogen deposition and its interactions with CO_2_ fertilization providing secondary drivers. However, this *transient* CO_2_ fertilization hypothesis contradicts the observed behavior of the residual land sink, which seems to experience a step increase from ~2000 onward (Le Quéré et al., 2016).

Furthermore, variations in climate have a strong influence on carbon cycling (Friedlingstein et al., 2006), and we simulated a net loss of carbon due to surface warming since the turn of this century. However, the impact of climate on the recent behavior of the land sink is relatively uncertain (Friedlingstein, 2015; Mystakidis et al., 2016). The findings of this study are contrary to the conclusions of Ballantyne et al. (2017), who argue that relatively cool surface temperatures (over 1998–2012—warming hiatus) reduced soil respiration, inducing a carbon sink. However, this warming hiatus hypothesis itself has been called into question because the changes in seasonal land sink trends between warming (1982–1998) and hiatus (1998–2014) periods do not match the changes in seasonal temperature trends (Zhu et al., 2018), and so changes in seasonal temperature are unlikely to be drivers of reduced annual ecosystem respiration. Thus, following contradictory studies, the mechanism(s) behind the increased terrestrial carbon sink since 2000 remain elusive.

Although our results provide a useful indication of the competing factors controlling the land carbon sink over the historical period, there are a number of process simplifications and limitations in our modeling methods that need to be considered. For example, we do not consider the effects of LULCC in this study. However, LULCC emissions are used directly in calculating the residual land sink, and so any errors in LULCC emissions propagate through to the residual sink estimate. So, if the 21st century decline in LULCC emissions is underestimated (Andela et al., 2017; Kondo et al., 2018; Liu et al., 2015), the uptick in the residual sink will be overestimated, meaning our modeled results would be more in agreement with the *observed* sink.

Further, the low temporal resolution (decadal mean) of our nitrogen deposition driver data will mask to some extent any abrupt related changes in the coupled carbon‐nitrogen cycles. However, as our analysis is based on decadal scales, we are reasonably confident in capturing the main response to changing nitrogen deposition. Regarding changes in spatial patterns of nitrogen deposition in the recent period, Chinese and North American trends are well validated (Liu et al., 2013; Xing et al., 2015). The simulated trends in nitrogen deposition over Western Europe seem, however, less robust as suggested by a recent satellite study indicating opposing trends (Jia et al., 2016). This discrepancy can be reconciled as Jia et al. (2016) did not include the contribution from ammonia to the total deposition flux due to lack of observations, yet ammonia could be an important component of the changes in total nitrogen fluxes in agricultural and biomass burning regions (Warner et al., 2017). Nonetheless, as the large‐scale changes seem to be realistic, we are satisfied that we accurately capture recent changes in deposition.

Additionally, even though CLM4.5‐BGC simulates detailed carbon and nitrogen cycles, there are still a number of shortcomings associated with carbon‐nitrogen biogeochemistry schemes (Thomas et al., 2013). One example is the fixed C:N ratios for plant tissues, which prevents ecosystems adapting to new conditions. In situ studies have shown that ecosystems exhibit increasing C:N ratios under increasing CO_2_, enabling high carbon storage per unit nitrogen (Dybzinski et al., 2015; Finzi et al., 2006). Further, CLM4.5‐BGC does not account for the varying dynamics of above/below‐ground carbon allocation, whereby there is increased root allocation under elevated atmospheric CO_2_ and nitrogen stress. This process has been shown to mediate plant response to elevated CO_2_ levels (Drake et al., 2011).

Newer versions of CLM improve upon the formulation used in this study, by introducing dynamic allocation and a more sophisticated representation of plant nitrogen uptake (Ghimire et al., 2016). Finally, our model does not consider the considerable uncertainty caused by biological fixation (Cleveland et al., 1999), which provides a major input of new nitrogen to terrestrial ecosystems and which has been found to upregulate during periods when net carbon uptake rates are high (Batterman, Hedin, et al., 2013). Our model formulation scales biological nitrogen fixation to NPP, which does not accurately reflect the upregulation and downregulation that plants use in response to differences in nitrogen demand versus supply (Batterman, Hedin, et al., 2013; Batterman, Wurzburger, et al., 2013). Such an alternative modeling structure is recommended (Wieder et al., 2015). Furthermore, the carbon cost for acquiring nutrients, including from soil versus nitrogen fixation, is not currently simulated, and modeling studies have shown the importance of this in accurately simulating how plants respond to altered nitrogen availability (Brzostek et al., 2014; Shi et al., 2016).

## 5 Conclusion

Our results highlight the importance of synergistic effects between rising atmospheric CO_2_, nitrogen deposition, and a changing climate in regard to the evolution of the terrestrial carbon sink over the 20th century. However, with respect to the recent (post 2000) strengthening of the terrestrial carbon sink, our findings suggest that such synergistic effects between carbon, nitrogen and climate are not key factors because of the relatively small change in global nitrogen deposition over the last two decades. We find CO_2_ fertilization to be a main driver behind the increased carbon sink since 2000, in line with previous studies (e.g., Keenan et al., 2016), although the recently observed decline in the carbon sink across the Amazonian tropical forests suggest that another factor, such as nitrogen, may be limiting the size of the sink (Brienen et al., 2015; Hedin, 2015). Alternatively, variations in climate have the potential to drive changes in carbon storage. Our analysis suggests that climate variations weakened the carbon sink over the recent period. The response of the biosphere to recent variations in climate is, however, uncertain with conflicting conclusions about the magnitude of change in the post‐2000 carbon sink (Ballantyne et al., 2017; Zhu et al., 2018).

With signs of the warming hiatus ending (Fyfe et al., 2016) and the potential for increased nutrient limitation in the future with higher demand under enhanced CO_2_, along with the potential for reductions in nitrogen deposition in some regions (Kanakidou et al., 2016), it remains unclear how long the terrestrial carbon sink can continue to grow in line with fossil fuel emissions. Resolving this question is critical for resolving nutrient cycling and global change in the future.

## Supporting information



Supporting Information S1
