# Supplementary material for: Have Synergies Between Nitrogen Deposition and Atmospheric CO2 Driven the Recent Enhancement of the Terrestrial Carbon Sink?
Source: Global Biogeochem Cycles. 2019 Feb 10;33(2):163–80. doi: 10.1029/2018GB005922 (PMC6472506; doi:10.1029/2018GB005922)
Supplement: Supplementary file 1 — Supporting Information S1 [file GBC-33-163-s001.pdf]

AGU PUBLICATIONS

Global Biogeochemical Cycles

Supporting Information for

Have synergies between nitrogen deposition and atmospheric CO2 driven the recent enhancement of the terrestrial carbon sink?

Michael O'Sullivan[1*], Dominick V. Spracklen[1], Steve R. Arnold[1], Manuel Gloor[2], Sarah A. Batterman[2] and Wolfgang Buermann[1]

[1]Institute for Climate and Atmospheric Science, School of Earth and Environment, University of Leeds, Leeds, UK

[2]School of Geography, University of Leeds, Leeds, UK

*Corresponding author: Michael O'Sullivan
Institute for Climate and Atmospheric Science
School of Earth and Environment
University of Leeds, Leeds, LS2 9JT
United Kingdom
Email: eemos@leeds.ac.uk

Contents of this file

Figures S1 to S12
Table S1

Introduction

Supporting information contains figures and a table aiding explanation in main manuscript.

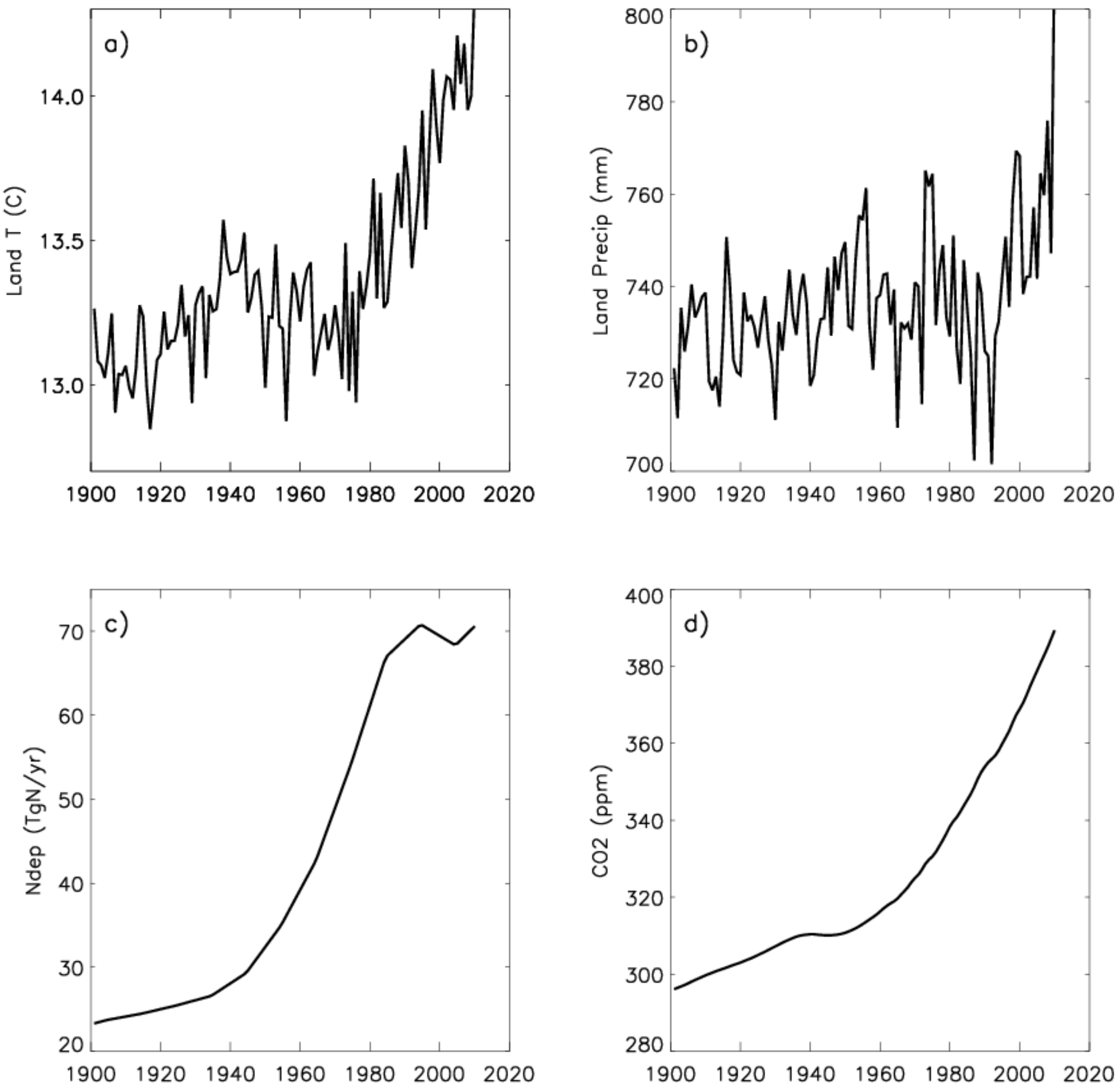

Figure S1 - Global mean changes in model drivers from 1901-2016 for a) land surface temperature (C), b) total precipitation over land (mm/yr), c) total atmospheric nitrogen deposition over land (TgN/yr), and d) atmospheric CO2 concentrations (ppm).

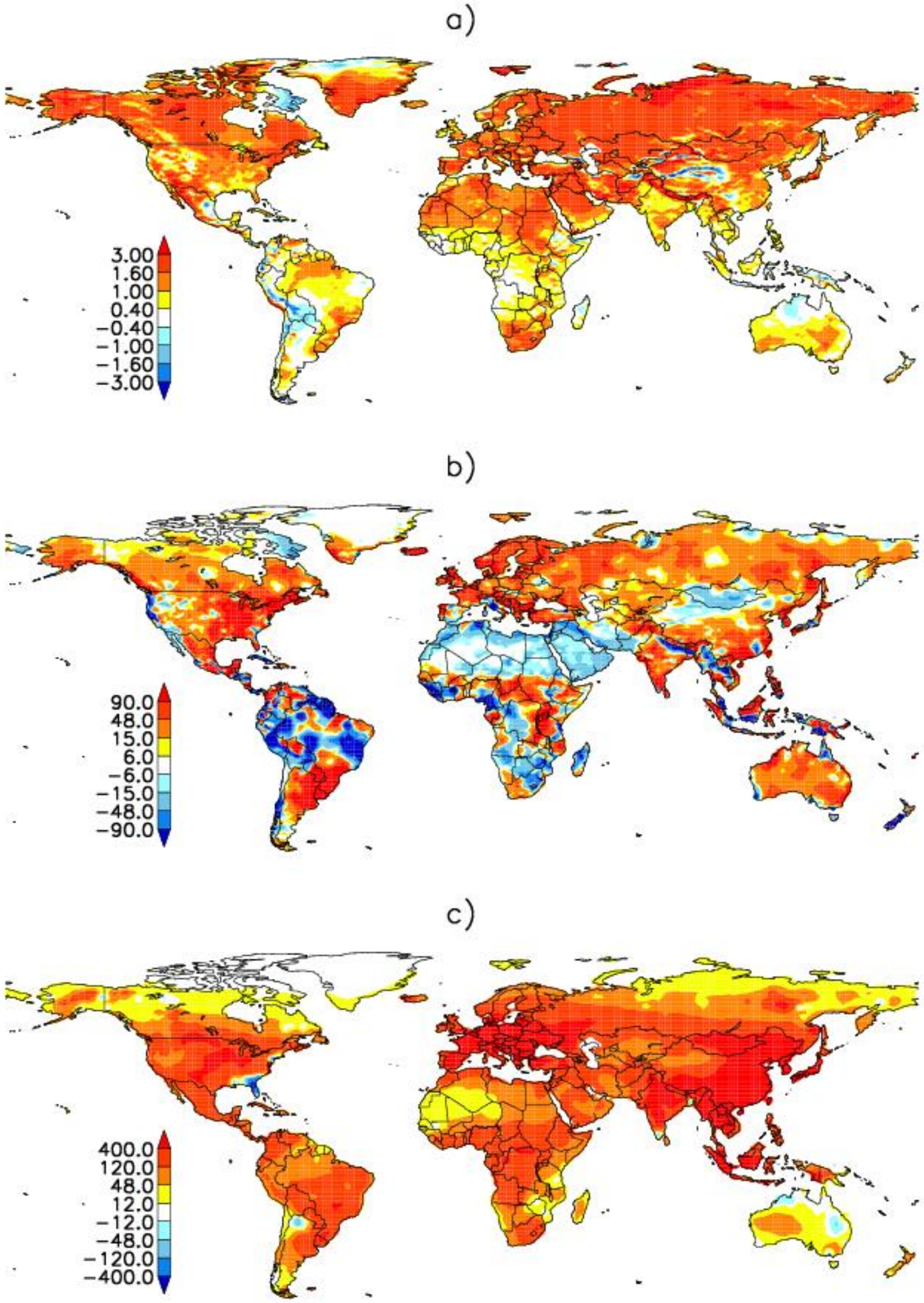

Figure S2 - Spatial trends in model drivers over the period (1901-1910) to (2007-2016) for a) land surface temperature (C), b) precipitation (mm/yr), and c) atmospheric nitrogen deposition (mgN/m2/yr).

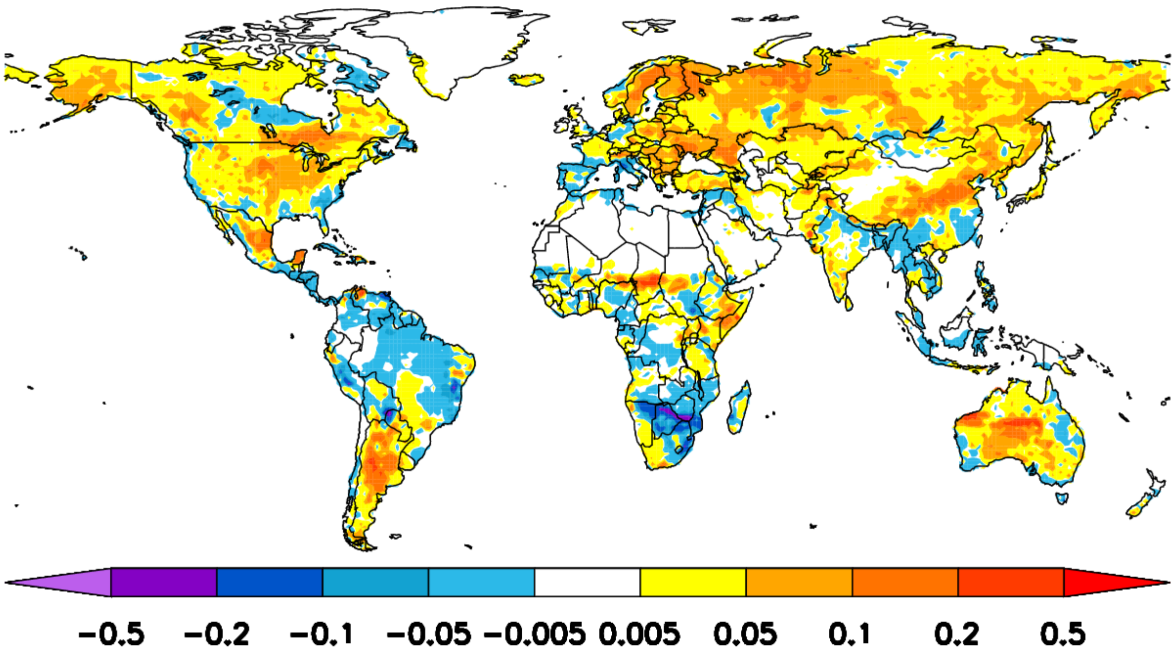

Figure S3 - Change in Btran between (1901-1910) and (2007-2016). Btran represents soil water availability in CLM and is a scaling factor (range 0 – 1) on stomatal conductance related to plant-available soil water.

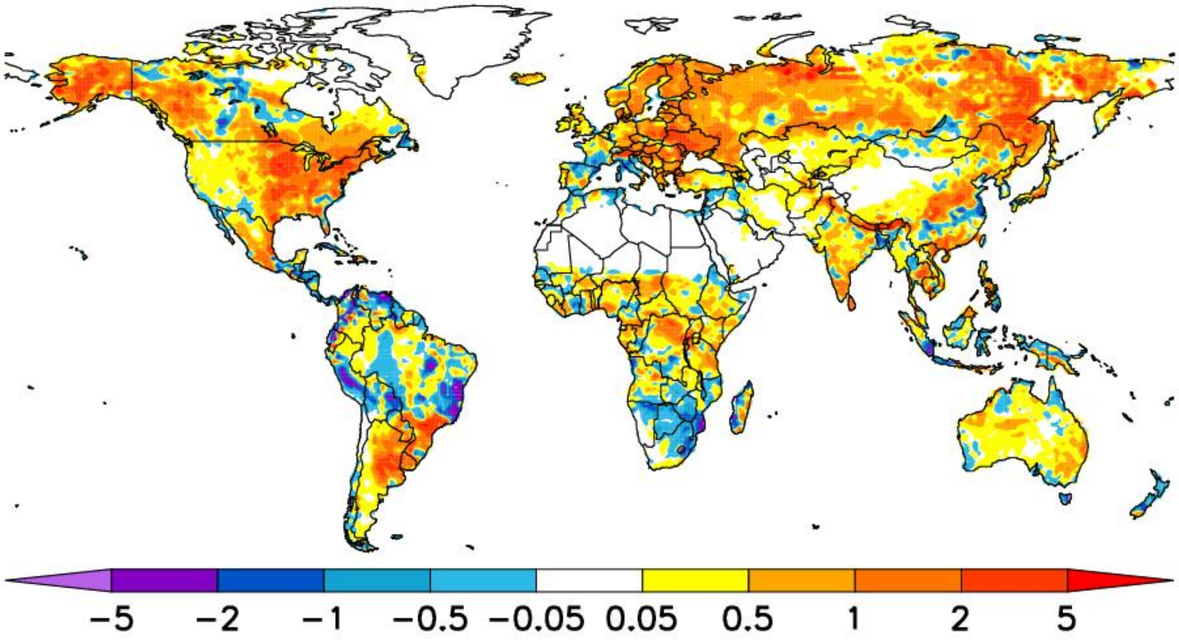

Figure S4 - Change in net nitrogen mineralisation (gN/m2/yr) between (1901-1910) and (2007-2016) due to climate forcing alone.

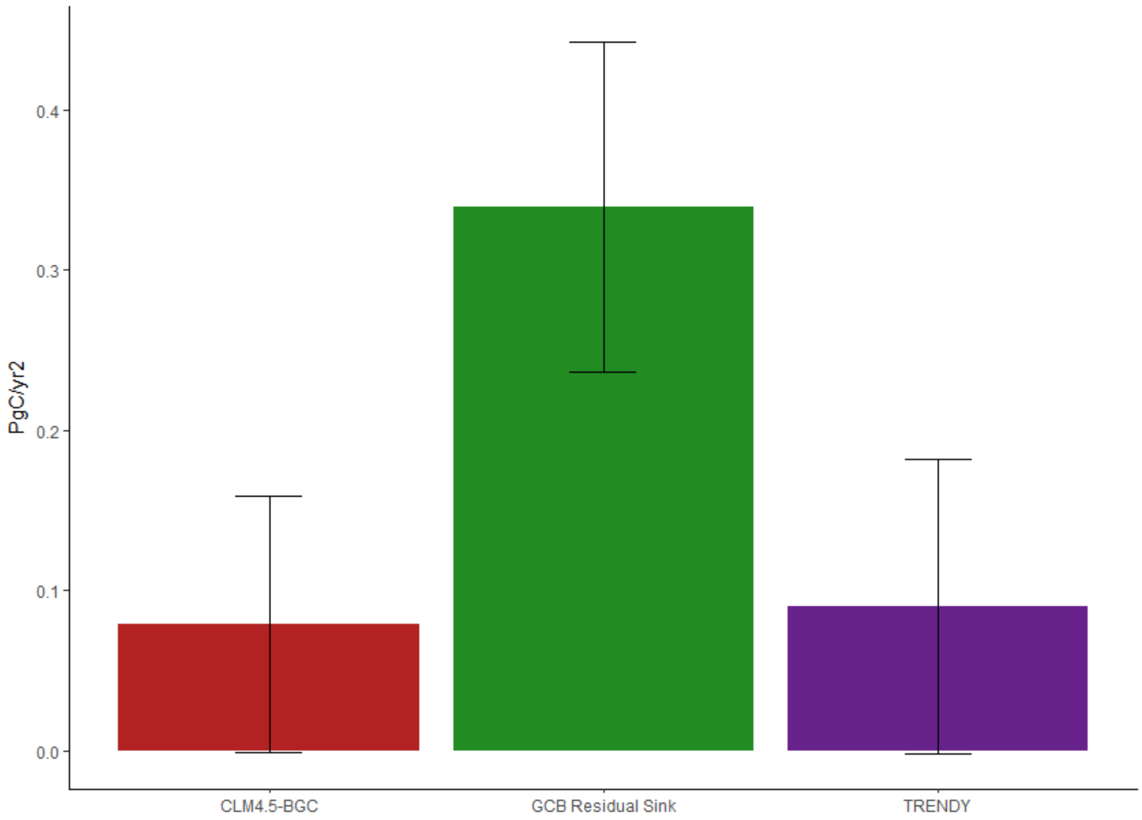

Figure S5 - Change in trend in land carbon uptake (PgC/yr^2) between 1990-2002 and 2002-2014. Changes in trend are shown for (this study) CLM4.5-BGC, the Global Carbon Budget residual sink (green), and TRENDY multi model mean (purple). The error bars for CLM4.5-BGC and GCB Residual Sink are calculated as the square root of the sums of squares of standard errors of trends for the two periods. The TRENDY error is calculated as the standard deviation of the change in trend across all models.

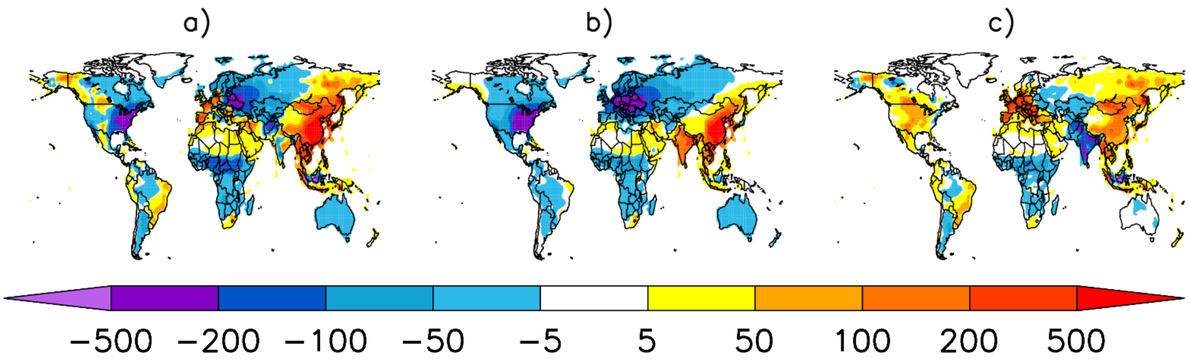

Figure S6 - Spatial maps of the change in nitrogen deposition (mgN/m2/yr) between 1990-1996 and 2010-2016 for a) total N deposition, b) NOy deposition, and c) NHx deposition. Since the early 1990s, global nitrogen deposition remained approximately constant (~80 TgN/yr), but there were large regional changes. Comparing the end (2010-2016) to the start (1990-1996) of this period, East Asia [75°E to 125°E and 10°N to 45°N] experienced a 27% increase in annual nitrogen deposition, mostly driven by fossil fuel (NOx) burning, but with a significant contribution from agricultural (NHx) activities (a-c). Reductions in fossil fuel burning in Europe [10°W to 25°E and 40°N to 60°N] and North America [120°W to 75°W and 30°N to 45°N] caused declines in deposition rates (b), however increasing agricultural intensity has countered the fossil fuel trend (c). Overall, the relative changes in nitrogen deposition in Europe and North America are smaller than East Asia, a 3% and 16% decrease, respectively.

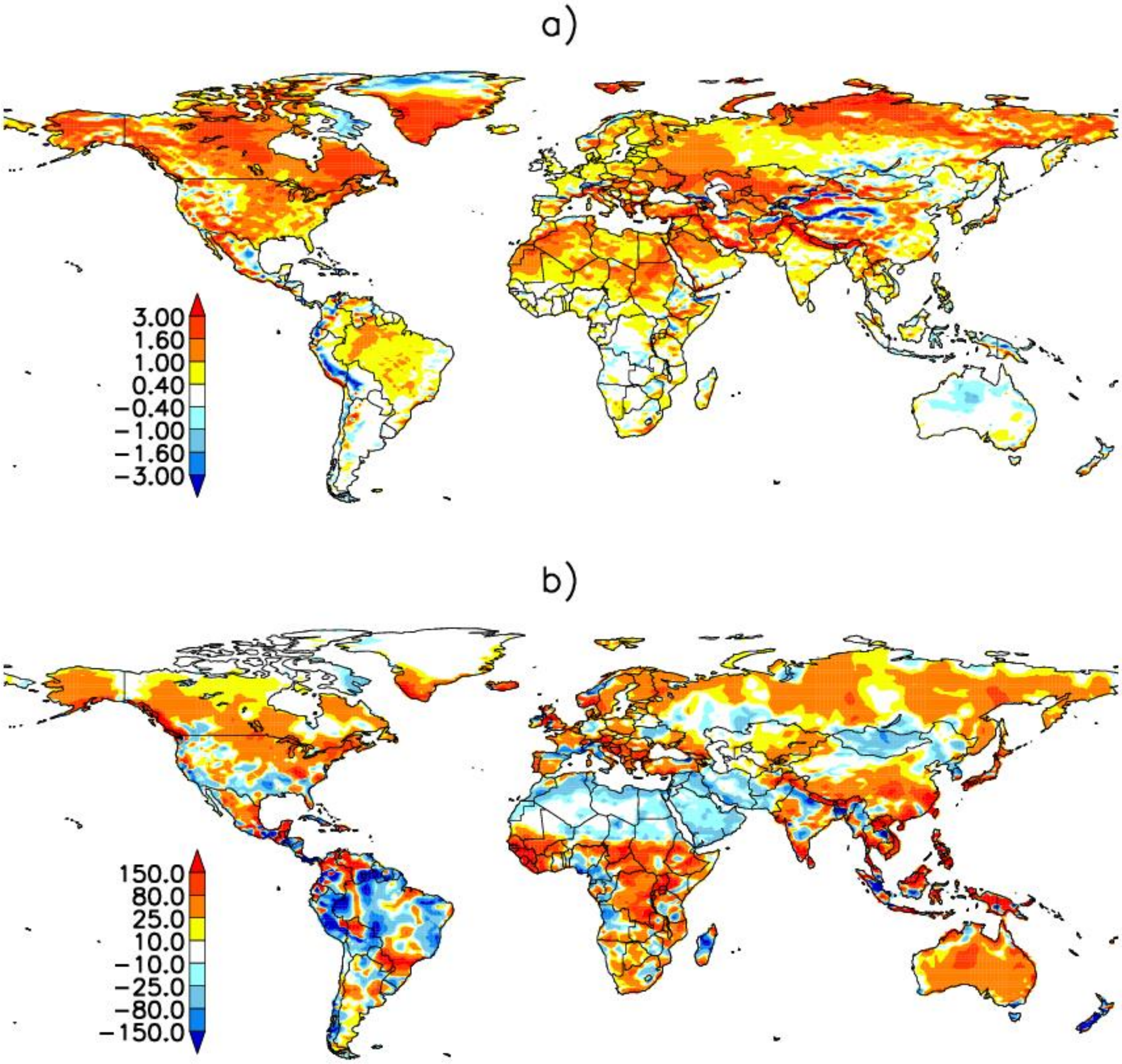

Figure S7 - Spatial trends in model drivers over the period (1990-1996) – (2010-2016) for a) land surface temperature (C), b) precipitation (mm/yr).

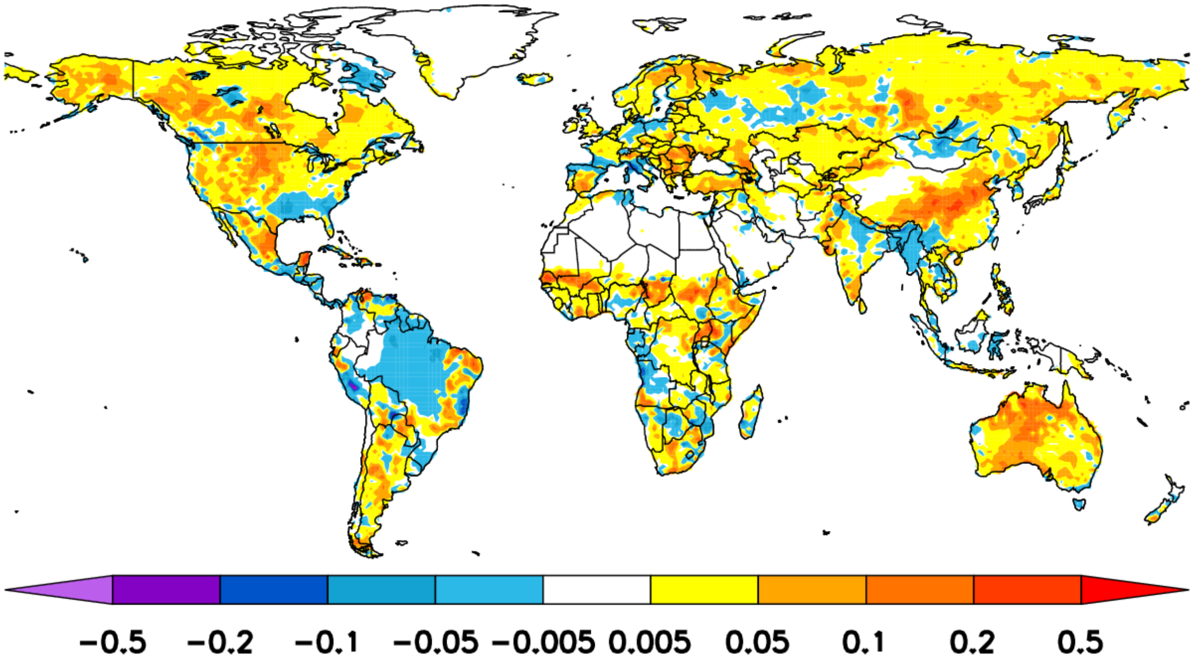

Figure S8 – Change in Btran between (1990-1996) and (2010-2016). Btran represents soil water availability in CLM and is a scaling factor (range 0 – 1) on stomatal conductance related to plant-available soil water.

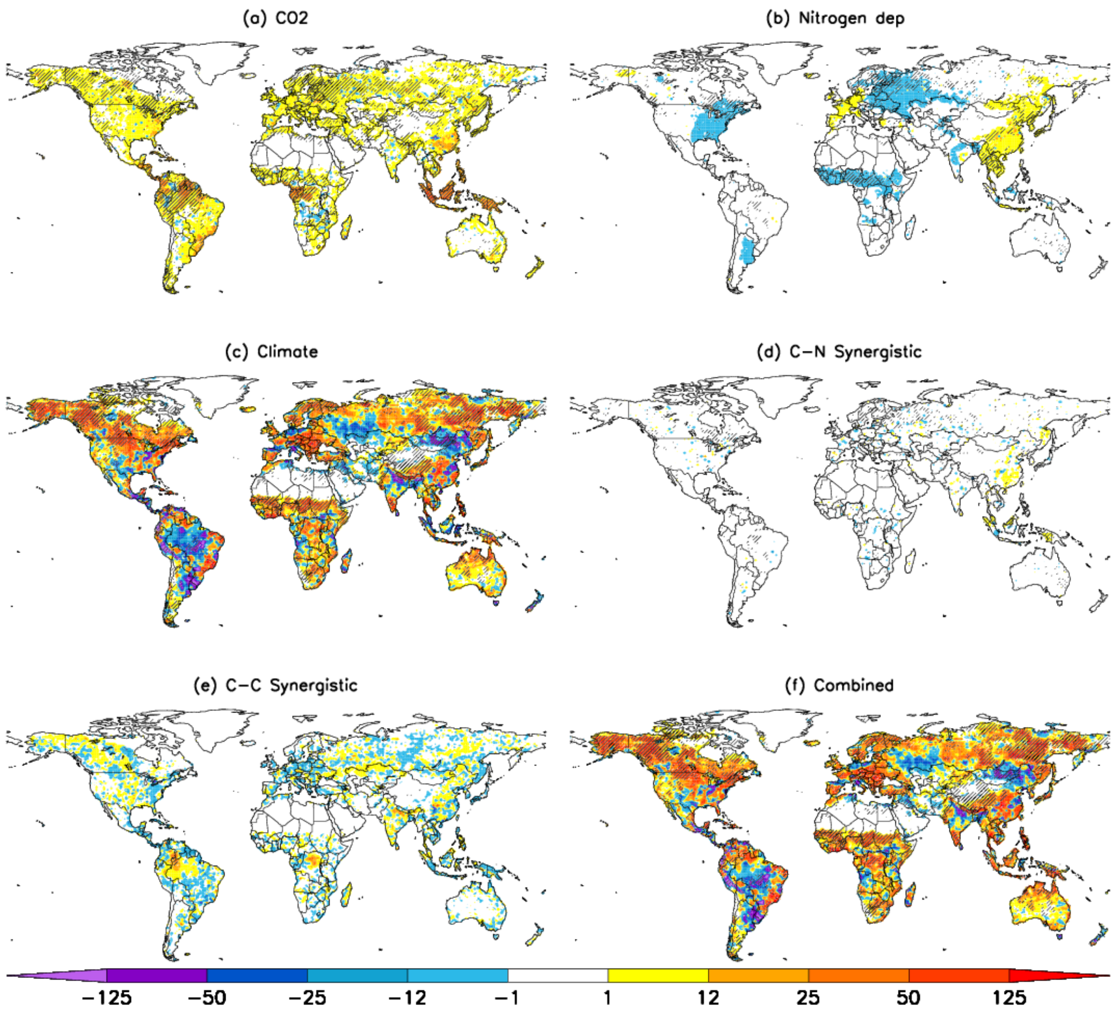

Figure S9 - Spatial patterns of heterotrophic respiration change (gC/m2/yr) due to a) CO2 fertilization, b) nitrogen deposition, c) climate, d) CN-synergy, e) CC-synergy, and f) combined effect. The patterns are based on a set of factorial simulations (see Methods). Respiration changes shown here are calculated as the difference between 2010-2016 and 1990-1996 mean values. Significant ($p<0.05$; Mann-Whitney U test) changes highlighted with hatching.

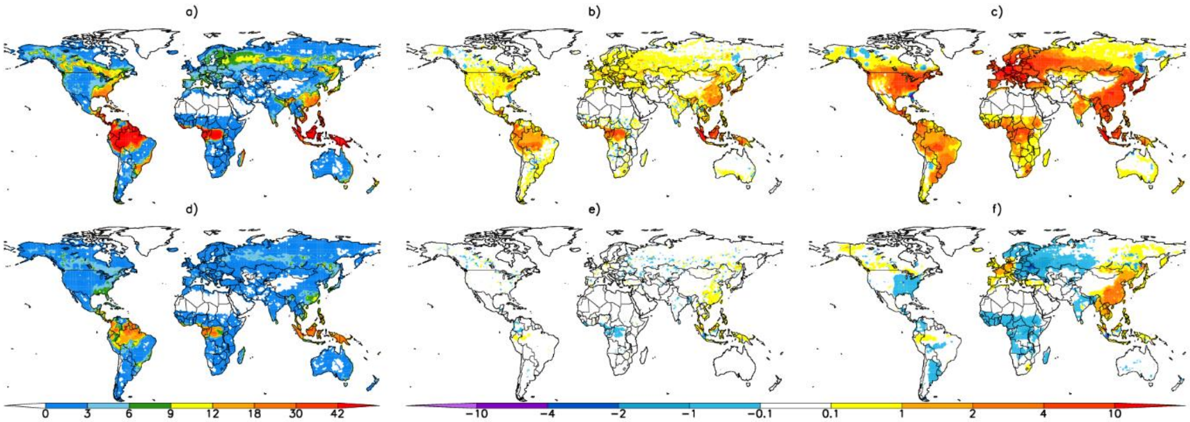

Figure S10 – Spatial distribution of Γ (gC/ppm) for the extended (top row) and recent (bottom row) periods calculated from the difference in total ecosystem carbon between the start and end of the study period (see Methods in main ms) for simulations with varying atmospheric CO2, constant climate and constant nitrogen deposition, (a,d). Panels b,e depict the influence of carbon-nitrogen synergy on Γ, and panels c,f depict the combined influence of nitrogen deposition and carbon-nitrogen synergy on Γ.

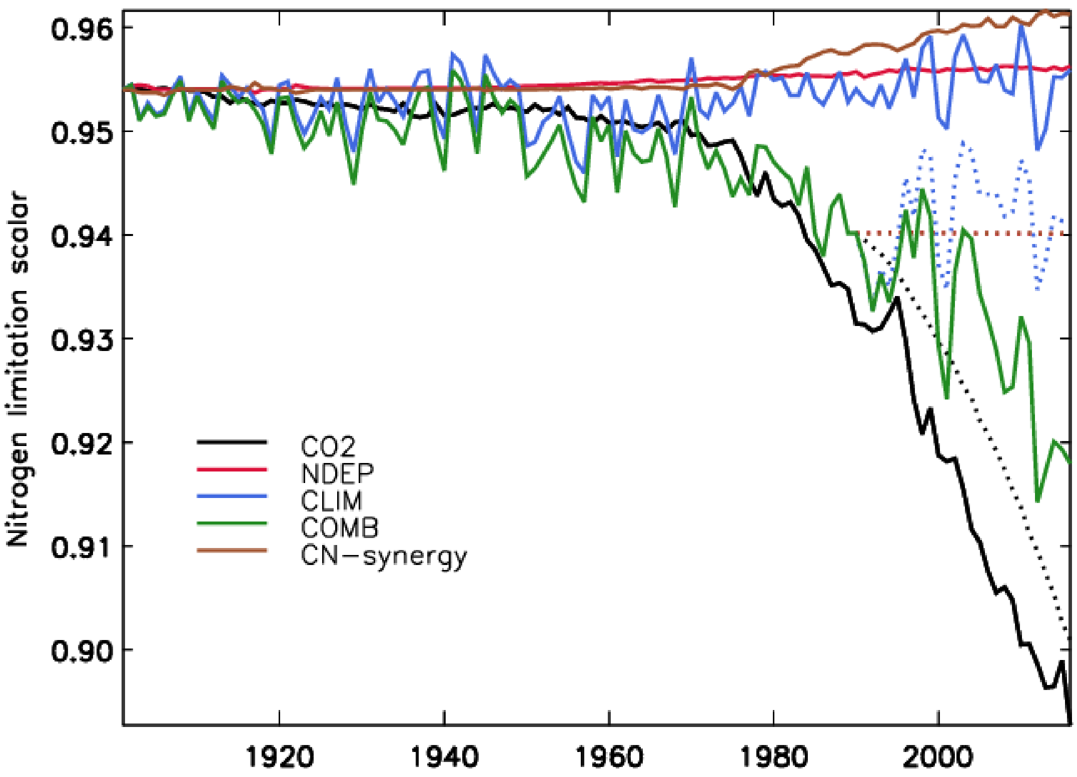

Figure S11 - Amazon nitrogen limitation scalar for 1901-2016 (solid) and 1990-2016 (dotted). Changes are relative to a control simulation with no variables changing. Contributions from CO2 (black), nitrogen deposition (red), climate (blue), combined (green), and CN-synergy (yellow) are shown. A decrease in the scalar value indicates higher nitrogen limitation (see Figure 8).

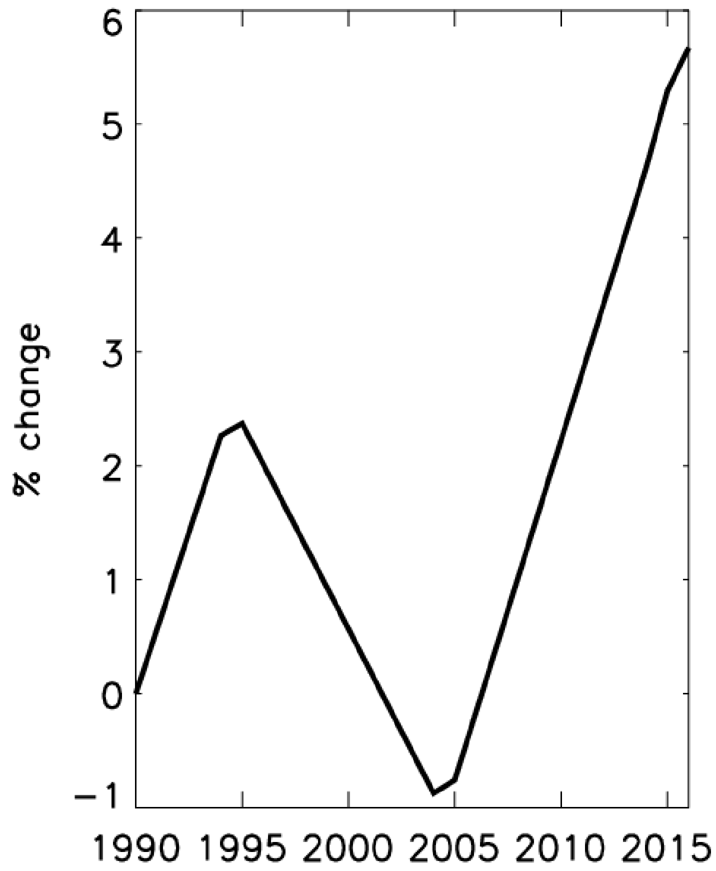

Figure S12 - Changes in global nitrogen deposition expressed as a percentage change from the start of the simulation (1990).

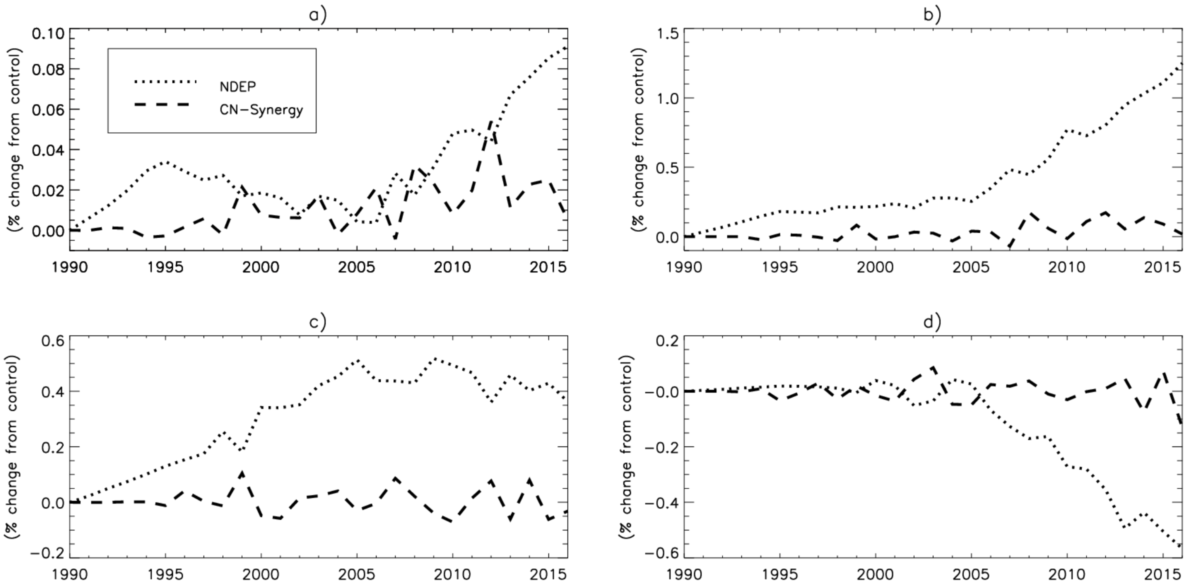

Figure S13 – Change (percentage from control run) in nitrogen limitation scalar from 1990 to 2016 using the recent simulations. The direct influence of nitrogen deposition (dotted) and carbon-nitrogen synergy (dashed) are shown. Four regions are shown, namely a) Global, b) East Asia (75°E to 125°E and 10°N to 45°N), c) Western Europe (10°W to 15°E and 40°N to 60°N), and d) North America (120°W to 75°W and 30°N to 45°N).

Region	ΔNdep (TgN/yr)	ΔNPP (TgC/yr)		ΔNBP (TgC/yr)	
		Ndep	CN-syn	Ndep	CN-syn
**Global**	1.78 (2.6)	30.9	13.3	33.0	3.0
**East Asia**	3.74 (26.6)	96.9	8.7	49.0	1.0
**East Europe**	-0.47 (-11.6)	-16.9	0.0	-6.6	0.5
**North America**	-0.89 (-16.2)	-19.7	-3.0	-10.9	-2.7
**West Europe**	0.22 (7.0)	9.2	-0.3	3.5	-0.6
**Central Africa**	-0.52 (-11.2)	-21.8	-5.6	-5.9	-4.4

Table S1 – Regional change in nitrogen deposition (Ndep) (TgN/yr) 1990-1996 to 2010-2016. Percentage change for Ndep shown in parenthesis. Changes in NPP (TgC/yr) and NBP (TgC/yr) due to the direct nitrogen deposition effect (Ndep) and the carbon-nitrogen synergistic effect (CN-syn) are also shown. Regions are defined as follows: East Asia (75°E to 125°E and 10°N to 45°N), East Europe (15°E to 40°E and 45°N to 70°N), North America (120°W to 75°W and 30°N to 45°N), West Europe (10°W to 15°E and 40°N to 60°N), and Central Africa (10°W to 30°E and 5°S to 10°N).
